# *Chinese wheat mosaic virus*‐derived vsiRNA‐20 can regulate virus infection in wheat through inhibition of vacuolar‐ (H^+^)‐PPase induced cell death

**DOI:** 10.1111/nph.16358

**Published:** 2020-01-07

**Authors:** Jian Yang, Tianye Zhang, Juan Li, Ne Wu, Guanwei Wu, Jin Yang, Xuan Chen, Long He, Jianping Chen

**Affiliations:** ^1^ State Key Laboratory for Managing Biotic and Chemical Threats to the Quality and Safety of Agroproducts Key Laboratory of Biotechnology in Plant Protection of MOA of China and Zhejiang Province Institute of Plant Virology Ningbo University Ningbo 315211 China; ^2^ State Key Laboratory Breeding Base for Zhejiang Sustainable Pest and Disease Control Zhejiang Provincial Key Laboratory of Plant Virology Institute of Virology and Biotechnology Zhejiang Academy of Agricultural Sciences Hangzhou 310021 China; ^3^ School of Forestry and Biotechnology Zhejiang Agriculture and Forestry University Hangzhou 310021 China

**Keywords:** Chinese wheat mosaic virus (CWMV), pathogenicity, vacuolar (H^+^)‐PPase (VP), virus‐derived small interfering RNA (vsiRNA), wheat

## Abstract

- Vacuolar (H^+^)‐PPases (VPs), are key regulators of active proton (H^+^) transport across membranes using the energy generated from PPi hydrolysis. The VPs also play vital roles in plant responses to various abiotic stresses. Their functions in plant responses to pathogen infections are unknown.
- Here, we show that TaVP, a VP of wheat (*Triticum aestivum*) is important for wheat resistance to *Chinese wheat mosaic virus* (CWMV) infection. Furthermore, overexpression of TaVP in plants induces the activity of PPi hydrolysis, leading to plants cell death.
- A virus‐derived small interfering RNA (vsiRNA‐20) generated from CWMV RNA1 can regulate the mRNA accumulation of *TaVP* in wheat. The accumulation of vsiRNA‐20 can suppress cell death induced by *TaVP* in a dosage‐dependent manner. Moreover, we show that the accumulation of vsiRNA‐20 can affect PPi hydrolysis and the concentration of H^+^ in CWMV‐infected wheat cells to create a more favorable cellular environment for CWMV replication.
- We propose that vsiRNA‐20 regulates *TaVP* expression to prevent cell death and to maintain a weak alkaline environment in cytoplasm to enhance CWMV infection in wheat. This finding may be used as a novel strategy to minimize virus pathogenicity and to develop new antiviral stratagems.

## Introduction

Plants have evolved sophisticated defense strategies to survive under biotic and abiotic stresses. These strategies include regulation of cellular pH value and ion homeostasis, important for various cell biology aspects. Optimal ion and pH gradients in cells are established and maintained by H^+^‐translocating enzymes (also known as H^+^‐pumps) and cation/H^+^ exchangers that are important for cell functions and plant development (Martinoia *et al.*, 2007; Rouached *et al.*, 2010). H^+^‐pumps use energy from hydrolysis of pyrophosphate (PPi) produced through ATP hydrolysis to support active proton transport across membranes (Maslowski & Kowalczyk, 1981; Martinoia *et al.*, 2007). In plant, there are three distinct membrane H^+^‐pumps that can generate pH gradients: the P‐type (H^+^)‐ATPase in plasma membrane to export H^+^ from cells (Serrano, 1993); the vacuolar (H^+^)‐ATPase (V‐ATPase) complexes that are encoded by ≥ 26 genes and can acidify vacuoles and other intracellular trafficking compartments (Strompen *et al.*, 2005); and the vacuolar (H^+^)‐PPases (VPs) that are single subunit proteins and can generate H^+^ gradients in endomembrane compartments using PPi (Zhen *et al.*, 1997). Expression of the *VP* gene can be upregulated by various abiotic stresses, including low nutrient, drought, cold, heat, heavy metal and salinity stresses (Gaxiola *et al.*, 2001; Han *et al.*, 2005; Queiros *et al.*, 2009; Kabala *et al.*, 2014; Wehner *et al.*, 2015; Graus *et al.*, 2018). Previous studies have shown that overexpression of *VP* genes can enhance drought and/or salinity stress tolerance in plants, including *Arabidopsis thaliana*, *Suaeda salsa*, wheat (*Triticum aestivum*) and *Nicotiana benthamiana* (Gaxiola *et al.*, 2001; Gao *et al.*, 2006; Guo *et al.*, 2006; Brini *et al.*, 2007; Graus *et al.*, 2018). Moreover, VP is known to play a vital role in vacuolar Na^+^ sequestration, auxin transport and auxin‐mediated growth (Parks *et al.*, 2002; Li *et al.*, 2005). To date, the functions of *VP* genes in pathogen, especially virus, infection in plants are largely unknown.

Plant viruses are obligate intracellular parasites and can cause diseases in plants, leading to significant losses in crop production. Plants employ multiple defense strategies to restrict viral infection, including hormone‐mediated defense responses, protein degradation, immune receptor signaling and regulation of metabolism (Liu *et al.*, 2017). RNA silencing also is an antiviral defense mechanism used by plants. This defense mechanism involves the recognition of viral double‐stranded RNAs (dsRNA) by host Dicer‐Like (DCL) enzymes and then the cleavage of viral dsRNAs into 21–24‐nt‐long small interfering RNAs (vsiRNAs) (Xie *et al.*, 2004). These vsiRNAs are then recruited by host Argonaute (AGO) proteins and guide the RNA‐induced silencing complexes (RISC) to target viral transcripts in a sequence‐specific manner (Margaria *et al.*, 2015; Carbonell, 2017). vsiRNAs also are known to cleave specific host homologous mRNAs to induce disease symptoms in *Cucumber mosaic virus* (CMV) Y‐satellite‐infected or in *Rice stripe virus* (RSV)‐infected plants (Shimura *et al.*, 2011; Shi *et al.*, 2016). Functional interactions between host mRNAs and vsiRNAs, and the vsiRNA‐guided cleavage of host mRNAs have rarely been studied experimentally. Therefore, the role of vsiRNA during virus infection in plants is still not fully understood. We speculate that some vsiRNAs generated during virus infection may create an unique cellular environment that is favorable for virus replication and/or movement between cells.

*Chinese wheat mosaic virus* (CWMV) has a bipartite single‐stranded positive sense RNA genome and is a member of the genus *Furovirus*, family *Virgaviridae* (Diao *et al.*, 1999; Adams *et al.*, 2009). CWMV RNA1 is 7147 nt long and encodes three proteins required for viral replication and movement. CWMV RNA2 is 3564 nt long and predicted to encode four proteins: the major capsid protein (CP, 19 kDa); two minor CP‐related proteins (N‐CP, 23 kDa and CP‐RT, 84 kDa) produced by initiation of translation at a noncanonical CUG start codon and by a read‐through strategy at the UGA termination codon, respectively; and a cysteine‐rich RNA‐silencing suppressor (CRP, 19 kDa) (Diao *et al.*, 1999; Andika *et al.*, 2013; Sun *et al.*, 2013a, b). Full‐length cDNA clones of CWMV RNA1 and RNA2 have been constructed and are infectious in both wheat and *N. benthamiana* plants (Yang *et al.*, 2016).

The interaction between host factors and viral components forms complicated networks. In the present study, we cloned and characterized a *VP* gene from wheat. Our results showed that during the early stage of CWMV infection in wheat, the virus can cause a significant induction of *VP* expression. Our results also showed that knockdown of *VP* expression in wheat enhances wheat susceptibility to CWMV infection significantly. By contrast, overexpression of *VP* in *N. benthamiana* leaves leads to a strong cell death phenotype and thus improve plant resistance to CWMV infection. Further analysis showed that the CWMV‐generated vsiRNA‐20 can mediate *TaVP* mRNA decay to suppress cell death, in a dosage‐dependent manner. Moreover, we have provided evidence through a reverse genetic technology which shows that the activity of PPi hydrolysis and cellular pH homeostasis are closely related to the expression level of vsiRNA‐20. In summary, this report provides evidence to show that vsiRNA is capable of regulating the expression of a host gene important for cell death to enhance virus infection. We reason that this finding may provide a new opportunity for the development of new antiviral strategies.

## Materials and Methods

### Virus source and plant growth conditions

The *Chinese wheat mosaic virus* (CWMV) used in the present study is from a source described previously (Yang *et al.*, 2016) and maintained in the laboratory until use. *Nicotiana benthamiana* plants were grown inside a growth chamber set at 25 ± 2°C, 70% relative humidity, and a 16 h : 8 h, light : dark photoperiod. Plants of wheat cv Yangmai 158 were grown inside a glasshouse and inoculated with CWMV at the four leaf‐stage. The inoculated wheat plants were grown inside a climate chamber set at 15 ± 2°C until evaluation.

### Plasmid constructions

Plasmids pCB‐35S‐R1 and pCB‐35S‐R2 contain a full‐length CWMV RNA1 or RNA2 sequence behind a 35S promotor (Yang *et al.*, 2016). Plasmids pCB‐T7‐R1 and pCB‐T7‐R2 contain a full‐length CWMV RNA1 or RNA2 sequence behind a T7 promotor (Yang *et al.*, 2016). For plasmid p35S:GFP‐UTR, primers P1F and P1R were used to amplify the 3′‐ untranslated region (UTR) of wheat *vacuolar H^+^‐pyrophosphatase* (*TaVP*) gene (GFP, green fluorescent protein). The resulting 196‐bp PCR product was double‐digested with the *Spe*I and *Sal*I restriction enzymes, and ligated into the *Spe*I/*Sal*I site in pCV1300:GFP for expression of a 3′‐UTR fused at its C‐terminus to GFP. For p35S:vsiRNA‐20, we used primers P2F and P4R to amplify osa‐micR528 and then used it as the template for the second PCR amplifications using primer P2F and P2R, P3F and P3R, or P4F and P4R (vsiRNA, virus‐derived small interfering RNA). The second round PCR products were mixed and used again as the template for the third PCR amplification using primer P2F and P4R. The resulting fragment was cloned into the pGEM‐Teasy vector (Promega), sequenced, released using enzyme *Bam*HI and *Sac*I, and inserted into the *Bam*HI/*Sac*I site in pCV1300 vector. For p35S:vsiRNA‐12, we used primers P13F and P15R to amplify osa‐micR528 and then used it for the second round PCR amplifications using primer P13F and P13R, P14F and P14R, or P15F and P15R. The resulting PCR products were mixed and used for the third PCR amplification using primer P13F and P15R, followed by insertion into the pCV1300 vector as described for p35S:vsiRNA‐20. For pCaMV35S:TaVP‐GFP, primer P5F and P5R were used to amplify the full‐length *TaVP*. This 2320‐bp PCR product was purified, double‐digested with enzyme *Nde*I/*Bam*HI, and ligated into the pCaMV35S:GFP vector. Plasmid p35S:OsVPE2‐RFP was constructed using the Gateway technology (Invitrogen). The first PCR was done using a rice cDNA and primer P16F and P16R. The second PCR was done using primers attB1 and attB2, and the first PCR product as the template. The resulting product was first cloned into the Gateway vectors and then the *Oryza sativa vacuolar Pi efflux transporters 2* gene (*OsVPE2*) fragment was transferred to the destination vector to produce p35S:OsVPE2‐RFP for expression of OsVPE2 fused at its C‐terminus to red fluorescent protein (RFP).

In order to conduct *Barley stripe mosaic virus* (BSMV)‐based virus‐induced gene silencing (BSMV‐VIGS), a 254‐bp fragment representing partial sequence of *TaVP* was reverse transcription PCR amplified using primers P6F and P6R. The resulting fragment was cloned, in a sense orientation, into the pCa‐γb vector (a gift from Zhensheng Kang, Northwest Agricultural and Forestry University, Yangling, Shaanxi Province, China) to generate pCa‐γb:TaVP. Plasmid pBSMV:TaPDS also was from Professor Zhensheng Kang. To transiently express TaVP in plant cells*,* we RT‐PCR amplified the full‐length *TaVP* gene using primers P7F and P7R. After double‐digestion with *Spe*I and *Sal*I enzymes, and the full‐length *TaVP* gene was inserted into the *Spe*I/*Sal*I site in pCaMV35S:HA to produce p35S:*Ta*VP‐HA. To construct pCB‐T7‐R1M, we PCR‐amplified the 5′ half of CWMV RNA1 (nucleotide position (nt) 1–2346, segment S1) from pCB‐T7‐R1 using primer P8F and P8R and then the 3′ half of CWMV RNA1 (nt 2367–7145, segment S2) using primer P9F and P9R. The S1 and S2 fragments were mixed and used as the template to amplify the full‐length CWMV RNA1 (segment S3) through overlap PCR using primer P8F and P9R. The S3 segment was double‐digested with *Bam*HI and *Sac*I enzymes and inserted into the *Bam*HI/*Sac*I site in vector pCB301, behind a T7 promoter. All of the plasmids were propagated in *Escherichia coli* DH5α cells and sequenced before use. All primers used in this study are listed in Supporting Information Table S1.

### RT‐PCR and Northern blot assays

Total RNA was extracted from plant tissues with TRIzol reagent (Invitrogen) and stored at −80°C until use. Integrity and concentration of each total RNA sample was checked using gel electrophoresis and a NanoDrop 1000 spectrophotometer (Thermo Fisher Scientific, Wilmington, DE, USA), respectively. First strand cDNA of each sample was synthesized using a First Strand cDNA Synthesis Kit (Toyobo, Kita‐ku, Osaka, Japan) and 1 μg total RNA per 20‐μl reaction. Quantitative PCR (qPCR) was conducted on an ABI7900HT Sequence Detection System (Applied Biosystems, Foster City, CA, USA) using an AceQ qPCR SYBR Green Master Mix (Vazyme, Nanjing, China). At least three biological replicates, with three technical replicates each, were used for each treatment and the results were analyzed using the 2^−ΔΔC*T*^ method (Livak & Schmittgen, 2001). Expression of the *actin* gene in each wheat sample was used as an internal control. Primers used for qRT‐PCR assays are listed in Table S1.

Northern blot assays were performed as described previously (Yang *et al.*, 2016). Briefly, 3 μg total RNA from a sample was loaded into a well in a 1.5% agarose gel with formaldehyde and separated through electrophoresis. The separated RNAs were blotted onto Hybond‐N^+^ membranes (Amersham Bioscience, Buckinghamshire, UK), cross‐linked for 2 h at 80°C, and the blots were probed with a DIG‐labeled DNA probe specific for the 3′‐terminus of CWMV RNAs. The DIG‐labeled probe was produced using a DIG High Prime DNA Labeling kit and the labeling was detected using the Detection Starter Kit II as instructed by the manufacturer (Roche). The final detection signal was captured using the Amersham Imager 600 (GE Healthcare Bio‐Sciences, Pittsburgh, PA, USA).

### 5′ Rapid amplification of cDNA ends (5′‐RACE) and cloning of the *TaVP* gene

The sequence information of the *TaVP* gene was obtained from the database of the National Center for Biotechnology Information (NCBI) with the accession no. XP_ MH643767. The full‐length sequence of *TaVP* was amplified by RT‐PCR using P10F/P10R primers and Super‐Fidelity DNA polymerase (Phanta Max; Vazyme Biotech Co. Ltd, Nanjing, China). The seedlings of wheat cv Yangmai 158 were used to extract total RNA by TRIzol reagent (Invitrogen). The cDNA generated and agarose gel electrophoresis was performed as described previously (Lu *et al.*, 2015). The purified PCR product was then inserted into the pGEM‐T Easy vector (Promega). The positive clones in *E. coli* were used to obtain the full sequence of *TaVP* identified by sequencing. Primers used for RT‐PCR are listed in Table S1.

5′‐RACE was performed as described (Shimura *et al.*, 2011) using the polyadenylated RNA fractions isolated from the total RNA samples with an Oligotex mRNA mini kit as instructed (Qiagen). Gene‐specific reverse primers for PCR and nested PCR were designed according to the sequence at the downstream of the cleavage sites in the *TaVP* sequence. The nested PCR products were separated in 2% agarose gels, purified using a DNA purification kit (Qiagen), cloned into the pGEM‐T Easy vector (Promega) and then sequenced. Primers used for 5′‐RACE are listed in Table S1.

### Western blot assay

Tissue samples were ground individually in liquid N_2_ and then homogenized in a protein extraction buffer (Sigma‐Aldrich) supplemented with Protease Inhibitor Cocktail Tablets (1 tablet/50 ml buffer; Roche). After 20 min centrifugation at 18 000 ***g*** at 4°C, supernatant was collected from each sample, boiled for 5 min and loaded into a well in SDS‐PAGE gels. Proteins were separated through electrophoresis and transferred to nitrocellulose membranes. Antibody specific for HA tag was purchased from Trans Gene (Beijing, China) and used to detect TaVP‐HA fusion protein in assayed plant tissues.

### Agro‐infiltration of *N. benthamiana* leaves

Plasmids were transformed individually into *A. tumefaciens* strain GV3101 by electroporation. Individual agrobacterium cultures were grown overnight, pelleted and re‐suspended in an induction buffer (1 M MgCl_2_ and 10 mM MES, pH 5.6, and 100 mM acetosyringone). After 3 h incubation at room temperature (RT), agrobacterium cultures were infiltrated individually into *N. benthamiana* leaves using needleless syringes. Three plants were infiltrated with an agrobacterium culture.

### Wheat protoplast isolation, transfection and confocal microscopy

Leaf blades and sheaths were collected from 2‐wk‐old wheat seedlings, cut to small pieces and submerged in a solution with 0.5 M mannitol, 1.5% cellulose RS (Yakult Honsa, Tokyo, Japan), 0.75 % macerozyme R10 (Yakult Honsa), 1 mM CaCl_2_ and 0.1 % BSA). The samples were incubated inside an incubator in the dark for 4–5 h at RT with 60 rpm shanking. Protoplasts were filtered through Miracloth (Millipore, Bedford, MA, USA), pelleted by 5 min centrifugation at 200 ***g***, and re‐suspended in a W5 solution containing 154 mM NaCl, 125 mM CaCl_2_, 5 mM KCl and 1.5 mM 2‐(*N*‐morpholino) ethane sulfonic acid (MES), pH 5.7. The protoplasts were pelleted again and re‐suspended in a MMg solution containing 0.4 M mannitol, 15 mM MgCl_2_ and 4.7 mM MES, pH 5.7. Plasmid DNA (10–20 μg DNA per plasmid) was added to protoplast solution, mixed with a 40% polyethylene glycol solution (40% PEG 4000, 0.4 M mannitol and 100 mM Calcium nitrate in distilled H_2_O), and incubated for 20 min at RT. The transfected protoplasts were rinsed twice with W5 solution, incubated overnight at RT, and examined under a Leica TCS SP8 confocal laser scanning microscope (Leica Microsystems, Heidelberg, Germany).

### BSMV‐based VIGS

BSMV RNAα, β and γ were *in vitro* transcribed from linearized plasmid DNAs (pBSMVα, pBSMVβ and pBSMVγ), respectively, using the mMessage mMachine T7 *in vitro* transcription kit as instructed (Ambion). The resulting BSMVα, β and γ RNA transcripts were mixed and diluted 20 times with RNase‐free H_2_O. For plant inoculation, 0.5 μl mixed BSMV RNAs was diluted with 9 μl inoculation buffer (1 ml 50 mM glycine and 50 mM K_2_HPO_4_, pH 9.2), and rub‐inoculated to the leaf 2 (bottom up) of a wheat seedling at the two‐leaf stage. Three plants were used for each treatment. Wheat seedlings inoculated with the inoculation buffer were used as mock‐inoculated controls. The inoculated seedlings were grown inside a dark growth chamber at 25 ± 2°C and high humidity for 24 h, and then under a 16 h : 8 h, light : dark photoperiod.

### Inoculation of wheat seedlings with CWMV RNAs

CWMV infectious RNAs were *in vitro* transcribed from linearized plasmid pCB‐T7‐R1, pCB‐T7‐R2 or pCB‐T7‐R1M as described previously (Yang *et al.*, 2016). The plasmids pCB‐T7‐R1, pCB‐T7‐R2 or pCB‐T7‐R1M were linearized with *Spe*I restriction digestion immediately downstream of the 3′ terminus of CWMV genomic RNA1, R1M and RNA2, and purified by repeated phenol/chloroform extractions and ethanol precipitation. The linearized plasmids were then used as DNA templates for *in vitro* transcription of CWMV RNA1, R1M and RNA2, respectively. The T7‐capped transcription reactions were performed with the Ambion Message Machine kit (Invitrogen) according to the manufacturer's protocols. After adding approximately 0.1 μg of linearized plasmid templates, the reaction mixtures were incubated at 37°C for 1 h and then 1 μl of reaction products were analyzed for the quantity and integrity of the transcripts by electrophoresis in a 1.0% agarose gel. Then, CWMV RNA1 or R1M transcript (5 µg) was mixed with 5 µg RNA2 transcript. The mixed RNA was resuspended in 1 ml inoculation buffer with a small amount of Celite, and mechanically inoculated to the leaf 2 (bottom up) of 2‐wk‐old wheat seedlings cv Yangmai158. The inoculated plants were grown inside a growth chamber set at 15 ± 2°C, unless stated otherwise.

### Enzyme activity assays

Tonoplast vesicles were isolated from wheat leaves through sucrose density gradient ultracentrifugation as described previously (De Michelis & Spanswick, 1986). Activities of vacuolar‐ (H^+^)‐PPase (VP) and vacuolar ATPase (V‐APTase) in the isolated tonoplast vesicles were measured using the method described previously (Smart *et al.*, 1998) with minor modifications. Hydrolysis of pyrophosphate (PPi) by VP was measured at 30°C in a reaction medium containing 1 mM Na_4_PPi, 50 mM KCl, 1 mM Na‐molybdate, 0.2% Triton X‐100, 1 mM MgSO_4_, 0.5 mM KF and 30 mM Tris‐MES, pH 7.2. ATP hydrolysis by V‐ATPase also was measured at 30°C in a reaction medium containing 1 mM NaN_3_, 0.1 mM sodiummolybdate, 0.5 mM sodium vanadate, 0.03% Triton X‐100, 3 mM ATP, 3 mM MgSO_4_ and 30 mM Tris‐MES, pH 7.2. The PPi hydrolysis activity was determined by the presence or absence of KCl or NaNO_3_. The nitrate‐sensitive and chloride‐stimulated activity was defined as the activity of V‐ATPase.

### Measurement of vacuolar pH

Changes of relative pH value in wheat protoplasts were determined using a cell‐permeable fluorescent indicator BCECF‐AM as described (Ferjani *et al.*, 2011). Briefly, protoplasts were pelleted after 5 min centrifugation at 100 ***g*** and resuspended in 300 µl of BCECF‐AM solution (20 µl BCECF‐AM reagent dissolved in 10 ml 1× assay buffer). Then 100 µl resuspended protoplasts were added into a well on a 96‐well plate. Three wells were used for each sample and the experiment was repeated three times. The plates were incubated at 37°C for 30 min inside a dark incubator supplemented with 5% CO_2_ followed by 30 min incubation at RT. 2′,7′‐bis (2‐carboxyethyl)‐5(6)‐carboxyfluorescein (BCECF) fluorescence was detected under a Leica TCS SP8 confocal laser scanning microscope (Leica Microsystems). The excitation wavelength was set at 488 or 440 nm and the emission was detected between 530 and 550 nm. The BCECF ratio (*F*_500_ : *F*_440_) images were generated using the ratio/concentration tool in the Leica Fluoview software and processed using imagej v.1.43 (http://rsb.info.nih.gov/nih-image/). The integrated pixel densities from the 488 nm‐excited images were divided by that from the 440 nm‐excited images to generate the BCECF *F*_500_ : *F*_440_ ratios. These ratios then were used to calculate the relative pH values from a calibration curve. For pH calibration, protoplasts were equilibrated in an equilibration buffer containing 50 mM BTP‐HEPES or MES, pH 5.0–8.4, and 50 mM ammonium acetate for 25 min. The samples were then examined under the confocal microscope as described above. Ten images from each of 30 analyzed protoplasts were taken, measured and used to determine the BCECF *F*_500_ : *F*_440_ ratios. The experiment was repeated at least five times.

### Statistical analysis

Each experiment reported in this study was repeated at least three times unless specifically noted otherwise. Data are presented as means ± SD three biological samples and each biological sample had at least three technical replicates. Student's *t*‐test was performed to determine the significant differences between control and treatment at each experiment. Significance values with *P* < 0.05, 0.01 or no significant difference were denoted with *, ** or ns, respectively.

## Results

### Cloning and phylogenetic analysis of *TaVP*

The *VP* gene was cloned from wheat through RT‐PCR. Sequencing results showed that the full‐length cDNA of *VP* gene is 2811 bp with a single open reading frame (ORF) of 2322 bp. Full‐length VP protein has a total of 774 amino acids (aa) with an estimated molecular mass of 80.3 kDa. Sequence alignment result showed that VP shares high aa sequence identities with its homologs in other plant species, including *Oryza sativa* VP 1 (OsVP1), *Brachypodium distachyon* VP 1 (BdVP1), *Zea mays* VP 1 (ZmVP1) and *Hordeum vulgare* VP 1 (HvVP1) (Fig. S1). Based on this finding, we refer to this wheat gene as *TaVP*. The sequence of this gene has been deposited at the NCBI database (GenBank accession no. MH643767).

**Figure 1:**
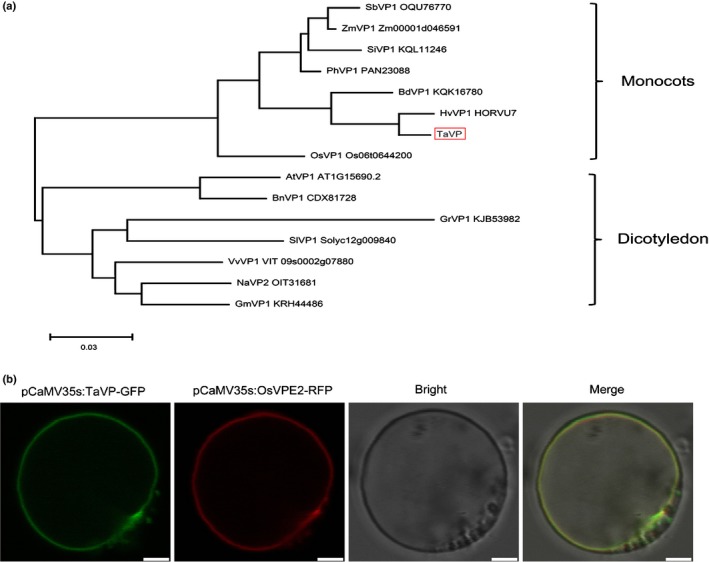
Sequence alignment using vacuolar (H^+^)‐PPase protein sequences from different plant species. (a) A phylogenetic tree constructed using the *Triticum aestivum* vacuolar (H^+^)‐PPase (TaVP) sequence and its closely related vacuolar (H^+^)‐PPase protein sequences from other plant species. TaVP is marked by a red box. The GenBank accession number of each protein is shown. The names of plant species are presented as: *Sorghum bicolor* (Sb), *Zea mays* (Zm), *Setaria italica* (Si), *Panicum hallii* (Ph), *Brachypodium distachyon* (Bd), *Hordeum vulgare* (Hv), *T. aestivum* (Ta), *Oryza sativa* (Os), *Arabidopsis thaliana* (At), *Brassica napus* (Bn), *Gossypium raimondii* (Gr), *Solanum lycopersicum* (Sl), *Vitis vinifera* (Vv), *Nicotiana attenuata* (Na), *Sorghum bicolor* (Sb), *Glycine max* (Gm). (b) Subcellular localization of TaVP protein. TaVP protein fused to green fluorescent protein (TaVP‐GFP) and *O. sativa* vacuolar Pi efflux transporters 2 protein fused to red fluorescent protein (OsVPE2‐RFP) were co‐expressed in wheat protoplasts under the control of the *Cauliflower mosaic virus* (CaMV) 35S promoter and observed under a confocal microscope. The photographs were taken in bright light for cell morphology, in dark field for green or red fluorescence, and in combination for yellow fluorescence. Bars, 50 µm.

In order to investigate the evolutionary relationship between TaVP and other VP proteins, we constructed a phylogenetic tree using aa sequences (Fig. 1a). The result showed that TaVP is most closely related to HvVP1. The closest TaVP homolog in *Arabidopsis* is VP 1 (AtVP1) and the closest homolog in *N. attenuata* is VP 2 (NaVP2) (Fig. 1a). This result suggests that TaVP has functions similar to that reported for other closely related VP proteins.

### Subcellular localization of TaVP in wheat cells

In order to determine the localization of TaVP in wheat cells, we co‐transfected wheat protoplasts with pCaMV35S:TaVP‐GFP and pCaMV35S:OsVPE2‐RFP, which expresses a rice vacuolar Pi efflux transporters 2 and RFP fusion protein (Xu *et al.*, 2019). At 2 d post co‐transfection, the protoplasts were examined for GFP and RFP fluorescence by confocal microscopy. Because the OsVPE2‐RFP is known to localize in vacuolar membranes, the co‐localization of TaVP‐GFP and OsVPE2‐RFP indicates that TaVP‐GFP is localized in the tonoplast too (Fig. 1b).

### Expression of *TaVP* in wheat is regulated by CWMV infection

**Figure 2:**
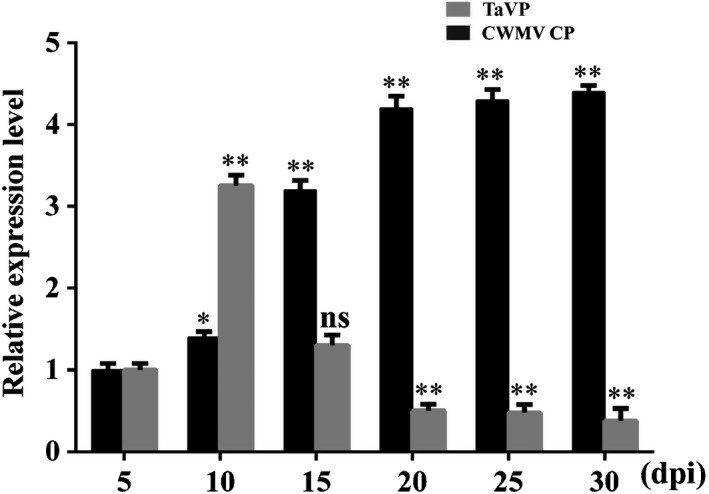
Expression of *Triticum aestivum* vacuolar (H^+^)‐PPase (*TaVP*) and accumulations of *Chinese wheat mosaic virus* (CWMV) in wheat at different numbers of d post‐inoculation (dpi) with virus. Expression of *TaVP* and accumulations of CWMV in CWMV‐inoculated wheat plants were determined by quantitative reverse transcription (qRT‐PCR) using *TaVP* or CWMV coat protein (CP) gene‐specific primers. The relative expression levels of *TaVP* or CWMV *CP* gene were calculated using the 2^−△△C^ *^T^* method. The expression level of the *TaActin* gene was used as an internal control. Each relative expression level is presented as mean ± SD from three biological samples and each biological sample had three technical replicates. Statistical analyses were done using Student's *t*‐test. *, *P* < 0.05; **, *P* < 0.01; ns, no significant difference.

Although several studies have indicated that overexpression of *VP* can enhance plant tolerance to drought and salinity stresses, the function of VP in plant response to pathogen infection remains unknown. In this study, the leaf 4 (bottom up) was selected and used to analyze the relative expression of *TaVP* in wheat plants after CWMV infection through RT‐qPCR. Our results showed that the accumulation level of CWMV CP was increased *c*. 4.5‐fold from 5 to 20 d post‐infection (dpi), and then maintained at this level until 30 dpi. The expression level of *TaVP* in the CWMV‐infected wheat plants was increased *c*. 3.2‐fold from 5 to 10 dpi, quickly declined at 15 dpi, and then maintained at a low level until 30 dpi (Fig. 2). This result indicates that the expression of *TaVP* in the CWMV‐infected wheat plants can be regulated, potentially to benefit CWMV infection in this host.

### Expression of *TaVP* affects CWMV accumulation in plant

**Figure 3:**
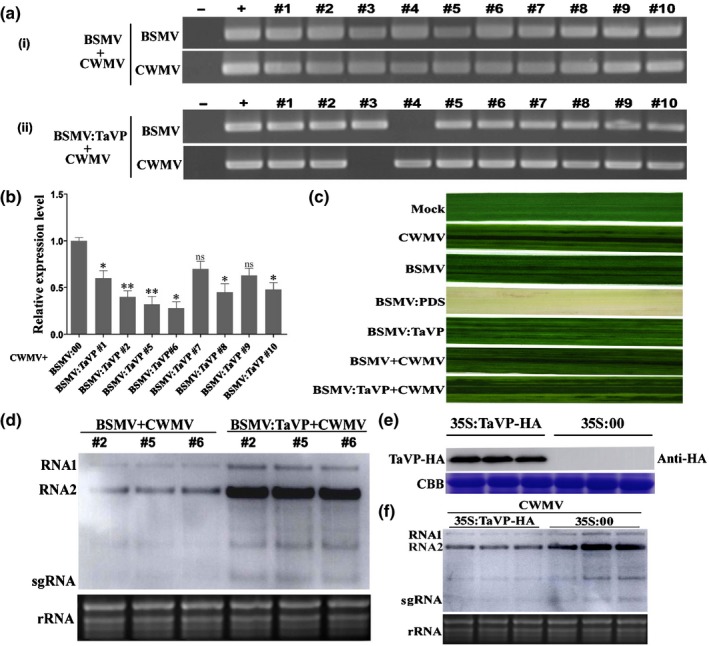
Effect of *Triticum aestivum* vacuolar (H^+^)‐PPase (TaVP) on *Chinese wheat mosaic virus* (CWMV) genomic RNA (gRNA) accumulation in wheat. (a) Reverse transcription PCR (RT‐PCR) detection of *Barley stripe mosaic virus* (BSMV) and CWMV infections in the BSMV + CWMV (i) or BSMV:TaVP + CWMV (ii) co‐inoculated wheat plants using BSMV RNAγ or CWMV RNA2 specific primers. The RT‐PCR products were visualized in agarose gels. Ten plants were analyzed for each treatment. Total RNA from a mock‐inoculated wheat plant was used as a negative control (−). Diluted plasmid pCB‐γ and pCB‐T7‐R2 were used as the positive control (+) for BSMV and CWMV, respectively. (b) Relative expression levels of *TaVP* in eight wheat plants co‐infected with CWMV and BSMV:TaVP. Total RNA from a BSMV and CWMV co‐infected wheat plant was used as a control. Each relative expression level is presented as mean ± SD from three biological samples and each biological sample had three technical replicates. Statistical analyses were done using Student's *t*‐test. *, *P* < 0.05; **, *P* < 0.01; ns, no significant difference. (c) Phenotypes in the fourth leaves of the plants inoculated with phosphate buffered saline (PBS) as Mock, CWMV, BSMV, BSMV:PDS, BSMV:TaVP, BSMV + CWMV and BSMV:TaVP + CWMV, respectively. Photographs were taken at 14 d post‐inoculation (dpi) with virus. (d) Detection of CWMV gRNAs in the fourth leaves of the plants co‐inoculated with BSMV + CWMV or BSMV:TaVP + CWMV through a Northern blot assay at 14 dpi. The ethidium bromide stained gel was used to show RNA loadings. Each treatment had three plants. (e) Expression of TaVP in the agro‐infiltrated *N. benthamiana* leaves were determined by Western blot using an HA‐specific antibody. Coomassie brilliant blue (CBB) stained rubisco gel was used to show the protein loadings. (f) Detection of CWMV gRNAs in the leaves co‐infiltrated with p35S:TaVP‐HA and the CWMV infectious clone or p35S:00 and the CWMV infectious clone through Northern blot at 4 dpi. The ethidium bromide stained gel is used to show RNA loadings. Three plants were used to represent a treatment.

In order to investigate the relationship between *TaVP* expression and CWMV infection in wheat plants, we inoculated 10 wheat seedlings with an RNA transcript representing CWMV, BSMV, BSMV:TaVP, BSMV:PDS, BSMV + CWMV or BSMV:TaVP + CWMV. After 7 dpi, we analyzed CWMV and BSMV infections in each assayed seedling through RT‐PCR using a pair of primers specific for the CWMV *CP* gene or the BSMV *CP* gene (Fig. 3a). We also analyzed the silencing level of the *TaVP* gene in the BSMV:TaVP + CWMV co‐inoculated wheat seedlings through RT‐qPCR using *TaVP*‐specific primers. The result showed that the *TaVP* transcript level in the assayed plants #2, 5 and 6 were better silenced (*P* < 0.01) than other plants (Fig. 3b). In this study, we also observed that the wheat plants infected with BSMV:TaPDS showed typical photo‐bleaching phenotypes in their systemic leaves by 14 dpi, whereas the plants infected with BSMV, BSMV + CWMV or BSMV:TaVP + CWMV showed mosaic symptoms (Fig. 3c). When total RNA samples isolated from the BSMV:TaVP + CWMV co‐infected plant #2, 5 and 6 were analyzed by Northern blot, we found that the level of CWMV genomic RNAs (gRNA) in the BSMV:TaVP + CWMV co‐infected plants was significantly higher than that in the plants (#2, 5 and 6) co‐infected with BSMV + CWMV (Fig. 3d). Because the expression level of *TaVP* was significantly induced in wheat plants at 10 dpi with CWMV, we decided to analyze the effect of *TaVP* overexpression on CWMV infection. Agrobacterium harboring p35S:TaVP‐HA was co‐infiltrated with CWMV infectious clones into leaves of *N. benthamiana* plants. Plants co‐infiltrated with an empty p35S:00 vector and CWMV infectious clones were used as controls. After confirming the expression of TaVP in *N. benthamiana* leaves by Western blot assays at 4 dpi (Fig. 3e), total RNA was extracted from each assayed plant for Northern blot assays. The result showed that much less CWMV gRNAs had accumulated in the leaves overexpressing TaVP, comparing the control leaves (Fig. 3f). Thus, we consider that CWMV infection is positively regulated by TaVP.

### Overexpression of *TaVP* increased PPi hydrolysis, leading to plant cell death

**Figure 4:**
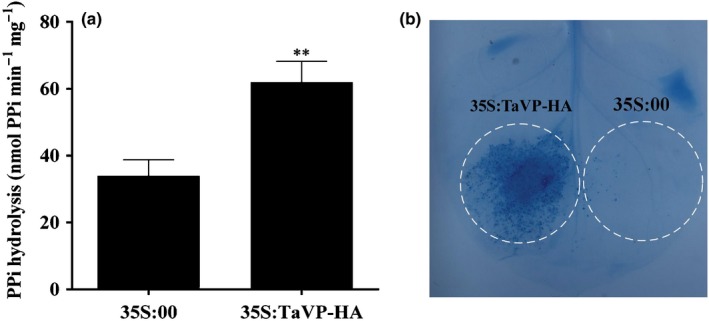
Overexpression of *Triticum aestivum* vacuolar (H^+^)‐PPase (TaVP) increases pyrophosphate (PPi) hydrolysis, leading to plant cell death. (a) Substrate hydrolysis by the vacuolar (H^+^)‐PPases (VPs) in crude membrane extracts from plants inoculated with 35S:00 or 35S:TaVP‐HA at 5 d post‐inoculation (dpi) with virus. Crude membrane extracts were prepared from leaves of > 10 plants and used for enzyme activity assays as described in the Materials and Methods section. Each result (mean ± SD) was from three biological samples and each biological sample had three technical replicates. Statistical analyses were done using Student's *t*‐test. **, *P* < 0.01. (b) At 5 dpi, the 35S:00 or 35S:TaVP‐HA‐infiltrated *Nicotiana benthamiana* leaves were detached and analyze for cell death through Trypan blue staining. The experiment was repeated at least three times with five or more plants per treatment.

Previous studies have shown that overexpression of VP in plant cells causes plasma membrane depolarization and then cell death due mainly to PPi hydrolysis rather than vacuolar acidification (Ferjani *et al.*, 2011; Graus *et al.*, 2018). To investigate the function of TaVP on PPi hydrolysis, we transiently overexpressed TaVP‐HA in *N. benthamiana* leaves through agro‐infiltration. After confirming the expression of TaVP‐HA in *N. benthamiana* leaves by Western blot assays at 3 dpi (Fig. S2), the activities of PPi hydrolysis in crude membrane fractions were measured. As expected, the activities of PPi hydrolysis in leaves overexpressing TaVP‐HA were significantly increased, compared to the leaves infiltrated with the empty vector (Fig. 4a). To test the function of TaVP on plant cell death, *N. benthamiana* leaves were agro‐infiltrated with plasmid p35S:TaVP‐HA or p35S:00 followed by Trypan blue staining at 5 dpi. Results showed that the leaf areas agro‐infiltrated with p35S:TaVP‐HA displayed strong cell death but not the leaf areas agro‐infiltrated with p35S:00 by 5 dpi (Fig. 4b), indicating that overexpression of TaVP can increase PPi hydrolysis, leading to plant cell death.

### vsiRNA‐20 from CWMV RNA1 targets the 3′‐UTR of *TaVP* mRNA

**Figure 5:**
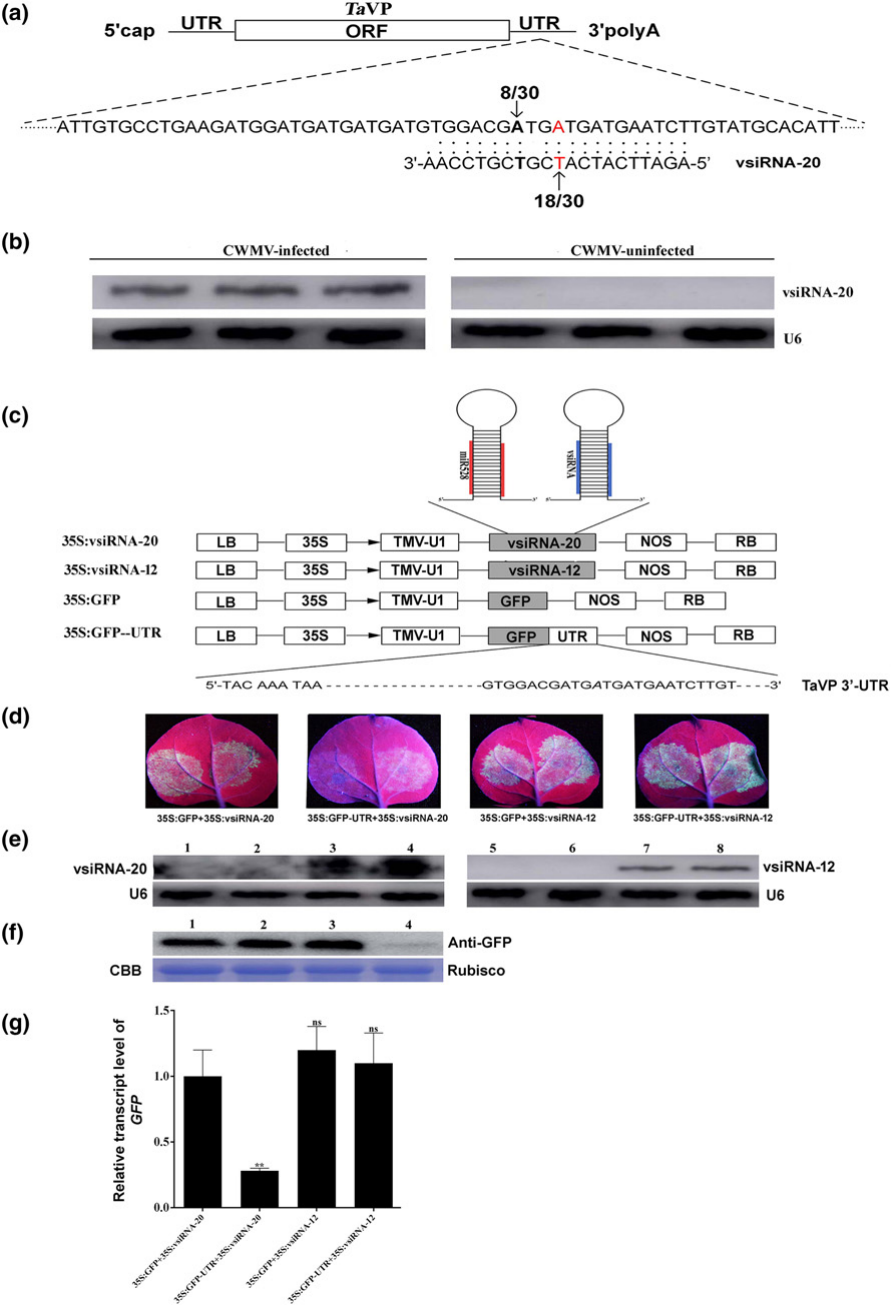
Targeting the 3′‐ untranslated region (UTR) of *Triticum aestivum vacuolar (H^+^)‐PPase* (*TaVP*) by virus‐derived small interfering RNA‐20 (vsiRNA‐20). (a) A schematic diagram showing the base pairing between vsiRNA‐20 and *TaVP*. The noncapped 5′ remnants of *TaVP* generated by vsiRNA‐20 cleavage were determined by 5′ rapid amplification of cDNA ends (5′‐RACE) using 30 selected clones. The frequencies of clones showing noncapped 5′ remnants are indicated. The predicted cleavage site is in red and the additional cleavage site is in black and bold. (b) Northern blot analysis of vsiRNA‐20 generated from *Chinese wheat mosaic virus* (CWMV) genome. Total RNA was extracted from three CWMV‐infected and three mock‐inoculated wheat plants. Expression of U6 was used as an internal control. (c) Schematic diagrams showing the structures of p35S:GFP, p35S:vsiRNA‐20, p35S:vsiRNA‐12 and p35S:GFP‐UTR, respectively. (d) Green fluorescent protein (GFP) fluorescence in *Nicotiana benthamiana* leaves co‐infiltrated with p35S:GFP + p35S:vsiRNA‐20, p35S:GFP‐UTR + p35S:vsiRNA‐20, p35S:GFP + p35S:vsiRNA‐12 or p35S:GFP‐UTR + p35S:vsiRNA‐12. The leaves were photographed under an UV light at 4 d post‐inoculation (dpi) with virus. (e) Detection of vsiRNA‐20 or vsiRNA‐12 accumulation in *N. benthamiana* leaves co‐infiltrated with (1) p35S:GFP + p35S:vsiRNA‐12, (2) p35S:GFP‐UTR + p35S:vsiRNA‐12, (3) p35S:GFP + p35S:vsiRNA‐20, (4) p35S:GFP‐UTR + p35S:vsiRNA‐20, (5) p35S:GFP + p35S:vsiRNA‐20, (6) p35S:GFP‐UTR + p35S:vsiRNA‐20, (7) p35S:GFP + p35S:vsiRNA‐12, and (8) p35S:GFP‐UTR + p35S:vsiRNA‐12 through Northern blot assays. Expression of U6 was used as an internal control. This experiment was repeated three times. (f) Detection of GFP in the *N. benthamiana* leaves co‐infiltrated with (1) p35S:GFP + p35S:vsiRNA‐20, (2) p35S:GFP + p35S:vsiRNA‐12, (3) p35S:GFP‐UTR + p35S:vsiRNA‐12, and (4) p35S:GFP‐UTR + p35S:vsiRNA‐20. Coomassie brilliant blue (CBB) large stained rubisco gel was used to show protein loadings. (g) Relative expression levels of *GFP* in *N. benthamiana* leaves co‐infiltrated with p35S:GFP + p35S:vsiRNA‐20, p35S:GFP + p35S:vsiRNA‐12, p35S:GFP‐UTR + p35S:vsiRNA‐12, and p35S:GFP‐UTR + p35S:vsiRNA‐20. Total RNA from a p35S:GFP + p35S:vsiRNA‐20 co‐infected leaves was used as a control. Each relative expression level is presented as mean ± SD from three biological samples and each biological sample had three technical replicates. Statistical analyses were done using Student's *t*‐test. **, *P* < 0.01; ns, no significant difference.

It was reported that vsiRNAs can target host gene mRNAs in cells (Shimura *et al.*, 2011; Shi *et al.*, 2016). Yang and colleagues previously analyzed vsiRNAs in CWMV‐infected wheat plants through deep sequencing (Yang *et al.*, 2014). To investigate whether the downregulation of *TaVP* expression was caused by the CWMV‐derived vsiRNAs, we compared *TaVP* sequence with the CWMV‐derived vsiRNAs that gave the highest numbers of reads (> 100) in the small RNA library (Table S2). Sequence comparison indicated that the 3′‐UTR of *TaVP* mRNA potentially could be targeted by vsiRNA‐20 (Fig. 5a). This finding prompted us to detect the expression of vsiRNA‐20 in wheat plants showing typical CWMV infection symptoms through Northern blot assays. Results of the experiment showed that vsiRNA‐20 was produced in the CWMV‐infected plants but not in the mock‐inoculated control plants (Fig. 5b). Consistent with this, qRT‐PCR results also showed that vsiRNA‐20 was detected in the samples of CWMV‐infected wheat plants, but neither in the samples of CWMV uninfected wheat plants nor a nontemplate water control sample (NTC) (Fig. S3). We then performed a 5′‐RACE using the same RNA sample used for the Northern blot assays followed by sequencing using the 5′‐RACE‐derived products. This study revealed two distinct cleavage sites in the 3′‐UTR of *TaVP* mRNA and 18 of the 30 sequenced clones had noncapped ends at the expected cleavage positions (Fig. 5a, red letters). Eight other clones showed different noncapped 5′‐ends upstream of the expected cleavage sites (Fig. 5a, bold black letters). To further confirm this finding, we constructed two artificial microRNA (amiRNA) vectors (i.e. p35S:vsiRNA‐20 and p35S:vsiRNA‐12) using *N. benthamiana* miR528 precursor as the backbone (Fig. 5c). vsiRNA‐12 (5′‐TTGGATACTGAACGACGCCTA‐3′) with reads > 100 localized in the 2460–2480 nt CWMV RNA2 negative genomic strand (Table S2) used as control. We also constructed a third amiRNA vector (p35S:GFP‐UTR) by ligating the 3′‐UTR of *TaVP* to the 3′‐end of a GFP gene (Fig. 5c). Plasmid p35S:GFP‐UTR and p35S:vsiRNA‐20, p35S:GFP and p35S:vsiRNA‐20, p35S:GFP‐UTR and p35S:vsiRNA‐12 or p35S:GFP and p35S:vsiRNA‐12 were co‐infiltrated into *N. benthamiana* leaves and the infiltrated leaves were examined for GFP fluorescence under an UV light at 4 dpi. Strong GFP fluorescence was observed in the leaves co‐infiltrated with p35S:GFP and p35S:vsiRNA‐20, p35S:GFP and p35S:vsiRNA‐12 or p35S:GFP‐UTR and p35S:vsiRNA‐12. By contrast, very weak GFP fluorescence was observed in the leaves co‐infiltrated with p35S:GFP‐UTR and p35S:vsiRNA‐20 (Fig. 5d). Northern blot results confirmed that vsiRNA‐20 and vsiRNA‐12 had accumulated in the leaves infiltrated with p35S:vsiRNA‐20 or p35S:vsiRNA‐12 (Fig. 5e). Western blot results also showed that much less GFP protein had accumulated in the leaves co‐infiltrated with p35S:GFP‐UTR and p35S:vsiRNA‐20 than the leaves co‐infiltrated with other plasmids (Fig. 5f). Consistent with this, qRT‐PCR results also showed that the transcript levels of *GFP* in the leaves co‐infiltrated with p35S:GFP‐UTR and p35S:vsiRNA‐20 were reduced significantly in comparison with the leaves co‐infiltrated with p35S:GFP and p35S:vsiRNA‐20. But the transcript leveld of *GFP* in the leaves co‐infiltrated with p35S:GFP and p35S:vsiRNA‐12 or p35S:GFP‐UTR and p35S:vsiRNA‐12 were not affected significantly (Fig. 5g). Thus, we conclude that vsiRNA‐20 can mediate *TaVP* mRNA decay in the plant cell.

### vsiRNA‐20 promotes CWMV accumulation in wheat plants

**Figure 6:**
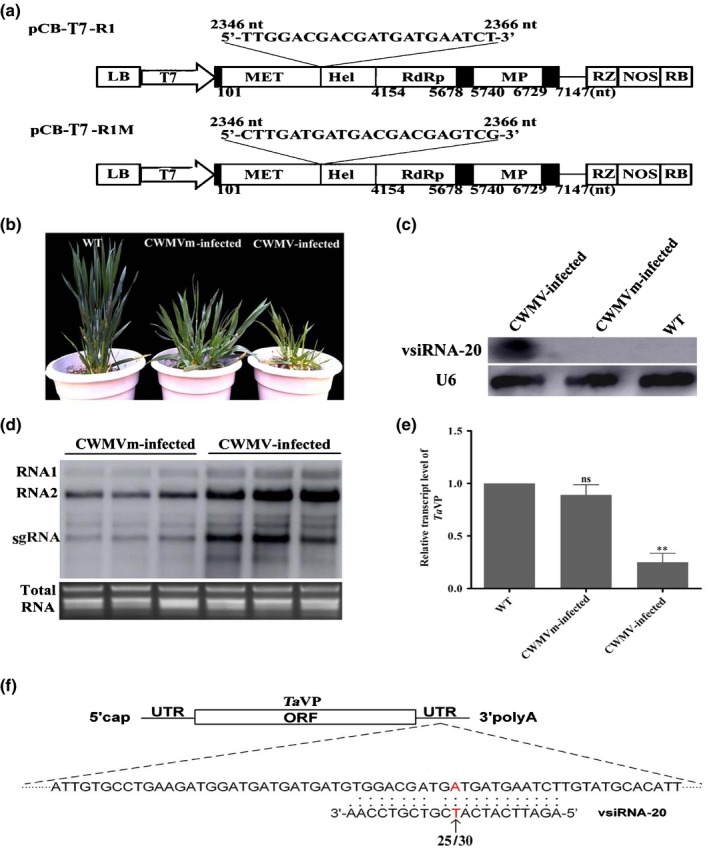
Influence of virus‐derived small interfering RNA‐20 (vsiRNA‐20) on *Chinese wheat mosaic virus* (CWMV) gRNA accumulations in wheat. (a) Schematic diagrams showing the construction of the pCB‐T7‐R1M mutant from plasmid pCB‐T7‐R1. (b) Phenotypes of wheat plants inoculated with phosphate buffered saline (PBS) buffer as wild‐type (WT), CWMV or its mutant (CWMVm). The plants were photographed at 98 d post‐inoculation (dpi) with virus. (c) Detection of vsiRNA‐20 accumulation in systemic leaves of wheat plants inoculated with CWMV or CWMVm transcripts, or with PBS buffer as WT through Northern blot assays. The expression of U6 was used as an internal control. The experiment was repeated three times. (d) Detection of CWMV gRNAs in wheat plants inoculated with CWMV or CWMVm transcripts through Northern blot at 98 dpi. The ethidium bromide stained gel was used to show RNA loadings. (e) The expression level of *TaVP* in wheat plants inoculated with CWMV or CWMVm transcripts, or with PBS buffer (WT). This experiment was repeated three times. Standard errors of individual means are shown. Statistical analyses were done using Student's *t*‐test. **, *P* < 0.01; ns, no significant difference. (f) A schematic diagram showing the base pairing between vsiRNA‐20 and *TaVP* in wheat inoculated with CWMV. The noncapped 5′ remnants of *TaVP* generated by vsiRNA‐20 cleavage was determined by 5′ rapid amplification of cDNA ends (5′‐RACE) using 30 selected clones. The frequencies of clones showing noncapped 5′ remnants are indicated. The predicted cleavage site is in red.

In order to elucidate the function of vsiRNA‐20 during CWMV infection in wheat, we altered the CWMV RNA1 sequence in pCB‐T7‐R1 to eliminate the production of vsiRNA‐20. This mutant pCB‐T7‐R1M vector contains a CTTGATGATGACGACGAGTCG sequence, with synonymous substitutions, at the nucleotide position 2346–2366 in CWMV RNA1 (Fig. 6a). *In vitro* transcribed RNA transcripts from pCB‐T7‐R1 or pCB‐T7‐R1M were mixed with equal amounts of RNA transcripts from pCB‐T7‐R2, and inoculated to wheat seedlings. At 14 dpi, leaf 4 of each assayed plant was harvested and analyzed for CWMV infection through RT‐PCR using CWMV *CP* gene‐specific primers (Fig. S4). By 60 dpi, the CWMV‐infected plants showed severe mosaic, yellowing and stunting symptoms, whereas the plants inoculated with R1M and R2 transcripts (referred to as mutant CWMV or CWMVm) showed much less mosaic and stunting symptoms (Fig. 6b). Northern blot results indicated that vsiRNA‐20 had accumulated in the CWMV‐infected wheat plants but not in the wheat plants infected with CWMVm (Fig. 6c). In addition, Northern blot results showed that the accumulation of CWMV in the CWMVm‐infected plants was significantly reduced compared with the CWMV‐infected plants (Fig. 6d). Further analysis revealed that the transcript level of *TaVP* in the CWMV‐infected wheat plants was reduced significantly compared with the mock‐inoculated control plants. But the transcript level of *TaVP* in the CWMVm‐infected wheat plants was not affected significantly (Fig. 6e), indicating that vsiRNA‐20 can regulate the expression of *TaVP* in wheat and is a positive regulator of CWMV infection. Subsequently, we performed 5′‐RACE assays using the same RNA sample used for qRT‐PCR assays followed by sequencing using the 5′‐RACE‐derived products. The results showed that 25 of the 30 sequenced clones had noncapped ends at the expected cleavage positions in the sample of CWMV‐infected wheat plants (Fig. 6f, red letters), whereas no cleavage sites were detected at the expected cleavage positions in the sample of CWMVm‐infected wheat plants (data not show). Thus, we deduced that vsiRNA‐20 promotes CWMV infection most possibly through mediating *TaVP* mRNA decay.

### vsiRNA‐20 regulates PPi hydrolysis and pH in CWMV‐infected wheat cells

**Figure 7:**
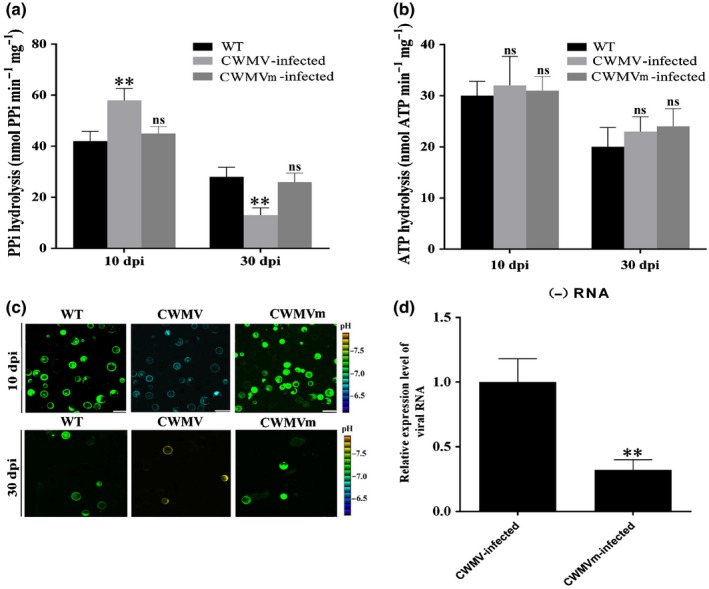
The vacuolar (H^+^)‐PPases (VPs) and the vacuolar (H^+^)‐ATPase (V‐ATPase) activities, and intracellular pH values in wheat protoplasts transfected with *Chinese wheat mosaic virus* (CWMV) or its mutant (CWMVm). Substrate hydrolysis activity of VP (a) and V‐ATPase (b) in crude membrane extracts from plants inoculated with buffer (Mock), CWMV or CWMVm at 10 and 30 d post‐inoculation (dpi) with virus, respectively. Crude membrane extracts were prepared from > 30 plants and used for enzyme activity assays. (c) Intracellular pH values in wheat protoplasts transfected with buffer (Mock), CWMV or CWMVm transcripts determined using BCECF‐AM dye (*n* = 10). The fluorescence ratios are shown in pseudocolor images and the emission at the corresponding excitation wavelength is presented in grayscale. (d) The relative transcripts level of viral RNA in protoplasts inoculated with CWMV or CWMVm. Each relative transcripts level is presented as mean ± SD from three biological samples and each biological sample had three technical replicates. Statistical analyses were done using Student's *t*‐test. **, *P* < 0.01; ns, no significant difference.

Results shown in Fig. 6(b) indicate that vsiRNA‐20 is generated specifically in wheat plants infected with CWMV but not CWMVm. To investigate the influence of vsiRNA‐20 on TaVP function, we measured PPi hydrolysis activities in the crude membrane fractions from the mock‐inoculated, CWMV‐infected or CWMVm‐infected wheat plants at 10 and 30 dpi, respectively. The activity of PPi hydrolysis in the CWMV‐infected plants was significantly higher than that in the mock‐inoculated or the CWMVm‐infected plants at 10 dpi. As expected, this activity was decreased significantly in the CWMV‐infected plants, comparing that in the mock‐inoculated or CWMVm‐infected plants at 30 dpi (Fig. 7a). The PPi hydrolysis activity in the CWMVm‐infected plants was similar to that in the mock‐inoculated plants at both 10 and 30 dpi. When the substrate hydrolysis activity of V‐ATPase was measured, no significant statistical differences were found among the three treatments (Fig. 7b), indicating that vsiRNA‐20 can suppress the PPi hydrolysis activity by regulating the expression of *TaVP*.

VPs is known to facilitate the active H^+^ proton transport across membranes using the energy generated from PPi hydrolysis (Martinoia *et al.*, 2007). To evaluate the effect of vsiRNA‐20 on intracellular pH value changes, we measured relative intracellular pH values in wheat protoplasts inoculated with CWMV or CWMVm using BCFL‐AM at 10 and 30 dpi, respectively (Fig. S5). Stronger fluorescence indicates a higher pH value (i.e. lower H^+^ concentration). Results showed that the protoplasts infected with CWMV showed much less fluorescence (pH = 6.8) at 10 dpi compared with that from the mock‐inoculated protoplasts (pH = 7.3) or that from the protoplasts infected with CWMVm (pH = 7.2, Fig. 7c). At 30 dpi, however, the protoplasts infected with CWMV showed stronger fluorescence (pH = 7.8) than that from the mock‐inoculated protoplasts (pH = 7.4) or from the protoplasts infected with CWMVm (pH = 7.38).

In addition, to confirm the possible role of vsiRNA‐20 in CWMV replication, we conducted protoplast transfection assays. Protoplast were isolated from wheat and the transfected with CWMV and CWMVm, followed by qRT‐PCR to monitor viral negative sense (−) viral RNA accumulation at 48 h post transfection. The results showed that viral RNA levels in protoplasts inoculated with CWMVm was significantly decreased in 0.32‐fold changes than those in protoplast inoculated with CWMV at the time point examined (Fig. 7d). Thus, we consider that vsiRNA‐20 is likely to target TaVP to weaken its activity and to decrease the concentration of intracellular H^+^ and PPi hydrolysis activity to create a more favorable cellular environment for CWMV replication in virally infected wheat cells.

### vsiRNA‐20 can suppress cell death induced by overexpression of TaVP in a dosage‐dependent manner

**Figure 8:**
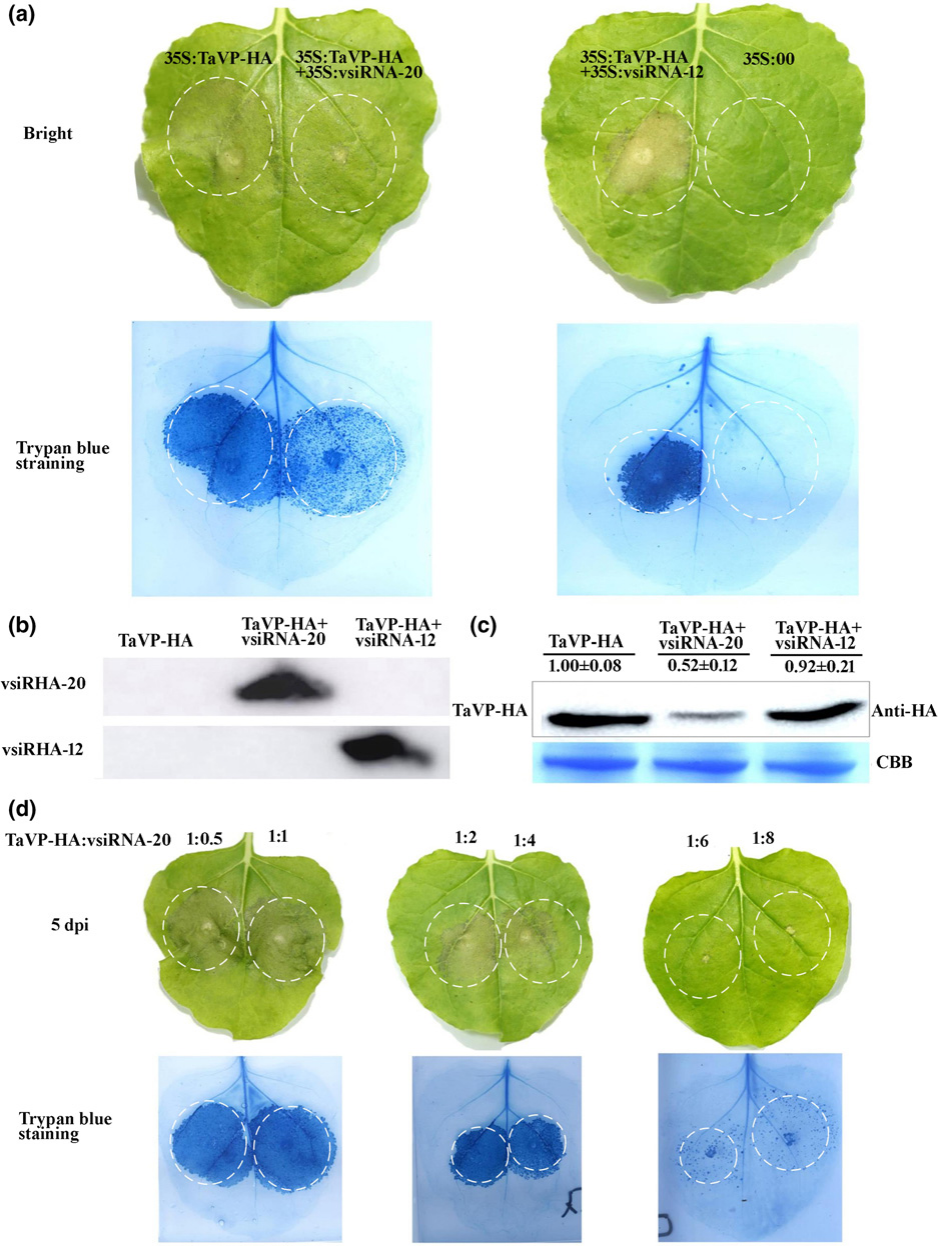
The virus‐derived small interfering RNA‐20 (vsiRNA‐20) can suppress cell death in *Nicotiana benthamiana* leaves overexpressing *Triticum aestivum* vacuolar (H^+^)‐PPase (TaVP). (a) At 5 d post‐inoculation (dpi) with virus, *N. benthamiana* leaves infiltrated with 35S:TaVP‐HA, 35S:00, 35S:TaVP‐HA + 35S:vsiRNA‐20 or 35S:TaVP‐HA + 35S:vsiRNA‐12 were detached, photographed and analyzed for cell death using Trypan blue staining. The experiment was repeated at least three times, using five or more plants per treatment. (b) Detection of vsiRNA accumulation by Northern blot assay. Total RNA was extracted from *N. benthamiana* leaves infiltrated with 35S:TaVP‐HA, 35S:TaVP‐HA + 35S:vsiRNA‐20 or 35S:TaVP‐HA + 35S:vsiRNA‐12 at 5 dpi. The resulting RNA samples were used for Northern blot assays. This experiment was repeated at least three times. (c) detection of TaVP‐HA in the *N. benthamiana* leaves infiltrated with 35S:TaVP‐HA, 35S:TaVP‐HA + 35S:vsiRNA‐20 or 35S:TaVP‐HA + 35S:vsiRNA‐12 through Western blot assays at 5 dpi. The Coomassie brilliant blue (CBB) stained large rubisco gel was used to show protein loadings. This experiment was repeated at least three times. (d) At 5 dpi, *N. benthamiana* leaves co‐infiltrated with 35S:TaVP‐HA and 35S:vsiRNA‐20 were detached, photographed and analyzed for cell death using Trypan blue staining. The concentration ratio (OD_600_) of 35S:TaVP‐HA/ 35S:vsiRNA‐20 was 1 : 0.5, 1 : 1, 1 : 2, 1 : 4, 1 : 6 and 1 : 8, respectively. The experiment was repeated at least three times, using five or more plants per treatment.

To investigate the effect of vsiRNA‐20 on plant cell death, we transiently co‐expressed TaVP‐HA and vsiRNA‐20 or TaVP‐HA and vsiRNA‐12 in *N. benthamiana* leaves through agro‐infiltration. *N. benthamiana* leaves agro‐infiltrated with p35S:TaVP‐HA or p35S:00 alone were used as controls. By 5 dpi, the regions expressing TaVP alone showed strong cell death but the regions co‐expressing TaVP‐HA and vsiRNA‐20 showed weak cell death (Fig. 8a). As expected, the regions co‐expressing TaVP‐HA and vsiRNA‐12 also showed cell death in this study (Fig. 8a). Although the regions expressing 35S:00 alone did not show any cell death (Fig. 8a). This result was confirmed by Trypan blue staining (Fig. 8a). Western blot assays using an anti‐HA antibody and Northern blot assays using a DIG‐labeled DNA probe specific for the 3′‐terminus of CWMV genomic RNAs further confirmed the above bio‐assay results (Fig. 8b,c). In addition, when the concentration of p35S:vsiRNA‐20 was increased during infiltration, the degree of cell death in the regions co‐expressing TaVP‐HA and vsiRNA‐20 was decreased (Fig. 8d). Thus, we conclude that vsiRNA‐20 can suppress cell death induced by overexpression of TaVP in a dosage‐dependent manner.

## Discussion

It is well‐known that the canonical role of plant VPs is to facilitate the sequestration of sodium (Na^+^) into vacuoles in plant. Functions of VP in many plant species, including *Arabidopsis thaliana*, *Suaeda salsa*, wheat (*Triticum aestivum*), tobacco and rice, have been studied extensively (Gaxiola *et al.*, 2001; Gao *et al.*, 2006; Guo *et al.*, 2006; Brini *et al.*, 2007; Zhang *et al.*, 2011; Graus *et al.*, 2018). Results of these studies indicate that VP is a protein with multiple roles, and one or more of these roles act(s) to improve plant defense against various abiotic stresses through enhancing plant development. However, the function(s) of VP in plant responses to biotic stresses have remained largely unknown. In this study, we cloned the *TaVP* gene from wheat and confirmed that this gene shares high sequence identities with homolog genes in other plant species (Fig. 1a,b). This result indicates that TaVP has functions similar to those reported for other plant species. Furthermore, knockdown of *TaVP* expression significantly increased Chinese wheat mosaic virus (CWMV) genomic RNA (gRNA) accumulation and disease symptom development, whereas transient overexpression of *TaVP* in *Nicotiana benthamiana* leaves resulted in a significant decrease of CWMV gRNA accumulation (Fig. 3). These results suggest that TaVP may play an important role in the interaction between the host and CWMV. Similar to other pathogen infections, plants also have been found to use innate pathogen‐associated molecular pattern (PAMP)‐triggered immunity (PTI) or activate effector‐triggered immunity (ETI) to limit viral infection (Korner *et al.*, 2013; Mandadi & Scholthof, 2013). One of the major mechanisms for plant antiviral immunity depends on RNA silencing, which often is suppressed by co‐evolving viral suppressors, thus enhancing viral pathogenicity in plants (Liu *et al.*, 2017). Here, we demonstrated that *TaVP* expression was significant induced at the early stage of CWMV infection in wheat (5–10 d post‐infection (dpi)) and was reduced at the virus progress infection stage (10–30 dpi) (Fig. 2). However, the CWMV capsid protein (CP) accumulation was increased after 5 dpi, peaked at 20 dpi and then maintained at this level until 30 dpi (Fig. 2). Thus, we speculate that *TaVP* may play a complicated role in the interaction between wheat and CWMV at different infection stages.

In this study, we conducted bioinformatic analyses using CWMV‐derived virus‐derived small interfering RNAs (vsiRNAs) and the full‐length *TaVP* gene sequence. Our results showed that vsiRNA‐20 can mediate the cleavage inside the 3′‐UTR of *TaVP* (Fig. 5a). We have determined through Northern blot assays that vsiRNA‐20 accumulated specifically in the CWMV‐infected wheat plants (Fig. 5b). The 5′‐RACE results confirmed the presence of *TaVP* transcripts with noncapped ends (Fig. 5c) and these noncapped ends matched the cleavage site inside vsiRNA‐20 (i.e. between nucleotides 10 and 11) (Elbashir *et al.*, 2001; Pantaleo *et al.*, 2007). In addition, a different cleavage site upstream of this one also was observed in this study (Fig. 5c). Cleavage at this site may be caused by cellular exonuclease activity after the vsiRNA‐20‐mediated cleavage (Gy *et al.*, 2007). Further studies using a green fluorescent protein (GFP) gene fused with the 3′‐UTR of *TaVP* showed that RNA transcript from this recombinant gene also was cleaved by vsiRNA‐20, even though the cleavage efficiency was lower than that for *TaVP* transcript (Fig. 5e–g). It is possible that there is a balance between *TaVP* transcription and endonucleotide cleavage activity, as reported for miRNA164a (Nikovics *et al.*, 2006). Previous studies have indicated that miRNA regulation of gene expression depends on the promoters that transcribe pre‐miRNAs and thus, stronger promoters often confer higher transcription efficiencies (Schwab *et al.*, 2006). Regardless of which explanation is correct, our results demonstrated that the 3′‐UTR of *TaVP* can be cleaved by vsiRNA‐20 *in vivo*. It is well‐known that the 3′‐UTR of host genes contains regulatory sequences capable of influencing gene expression (Barrett *et al.*, 2012). vsiRNAs have been shown to silence specific host mRNAs to inhibit virus accumulation in plants (Shimura *et al.*, 2011; Smith *et al.*, 2011). In the present study using CWMV inoculated with R1M and R2 transcripts (referred to as mutant CWMV or CWMVm) with synonymous substitutions also showed that the regulation of *TaVP* expression is vsiRNA‐20 specific (Fig. 6). Taken together, we propose that regulation of *TaVP* expression by vsiRNA‐20 is crucial for CWMV infection in wheat.

**Figure 9:**
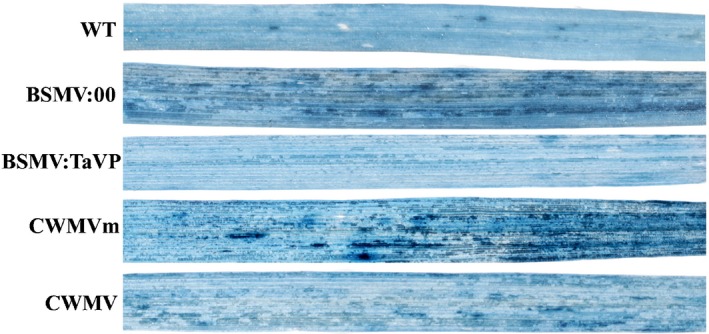
*Chinese wheat mosaic virus* (CWMV)‐infected wheat leaves were analyzed for cell death through Trypan blue staining. At 20 d post‐inoculation (dpi) with virus, wheat leaves inoculated with *Barley stripe mosaic virus* (BSMV:00), BSMV:TaVP, CWMV and its mutant (CWMVm) were detached, photographed and analyzed for cell death using Trypan blue staining. The experiment was repeated at least three times, using ≥ 10 plants per treatment.

Various proton (H^+^)‐pumps with different functions are known to localize in specific cellular components (Serrano, 1993; Segami *et al.*, 2018b). In this study, we analyzed TaVP subcellular location through a transient expression approach and showed that the TaVP‐GFP fusion is localized in the tonoplast (Fig. 1c). VP was reported to acidify vacuoles and other intracellular trafficking compartments in cells through generating H^+^ gradients in endomembrane compartments using pyrophosphate (PPi) (Strompen *et al.*, 2005). Comparing the mock‐inoculated wheat plants, the concentration of H^+^ in the CWMV‐infected wheat cells was increased (pH = 6.8) by 10 dpi and then decreased (pH = 7.8) by 30 dpi. In contrast, no significant changes of H^+^ concentration were observed in wheat cells infected with CWMVm between 10 and 30 dpi (Fig. 7c). Earlier reports had shown that *avian H5N1 influenza virus* (Reed *et al.*, 2010) and *Helicoverpa armigera stunt virus* (Penkler *et al.*, 2016) prefer higher concentrations of cellular pH. We propose that regulation of TaVP activity by vsiRNA‐20 is to disturb H^+^ gradients to maintain a weak alkaline environment in cytoplasm to promote CWMV replication in wheat plant. It is noteworthy that a different report had indicated that the major function of VP is to hydrolyze cytosolic PPi rather than vacuolar acidification in plant (Ferjani *et al.*, 2011). To investigate this possibility, we also analyzed the activity of PPi hydrolysis in this study. Our results showed that the PPi hydrolysis activity was significantly increased at the early stage of CWMV infection (10 dpi) and then decreased significantly by 30 dpi (Fig. 7a). Li and colleagues showed that suppression of VP activity increased V‐ATPase activity in *A. thaliana* (Li *et al.*, 2005). However, the V‐ATPase activities in the CWMV‐infected, CWMVm‐infected or mock‐inoculated wheat plants are similar (Fig. 7b). It is possible that V‐ATPase and VP play different roles in plant development (Krebs *et al.*, 2010). A more recent report indicates that the VP proton pump activity can adapt to cellular demands and the rapid increase of operating pump numbers lead to a cell death phenotype in *N. benthamiana* leaves (Graus *et al.*, 2018). Our results agree with this report and indicate that the PPi hydrolysis activity is higher in those plants overexpressing TaVP‐HA than that in the control plants (Fig. 4a). In addition, transient overexpression of TaVP in *N. benthamiana* leaves resulted in a cell death phenotype by 5 dpi (Fig. 4b). However, some studies also showed that overexpression of VP is sufficient for plants survival under certain conditions such as drought, salt and heat stresses (Li *et al.*, 2016; Segami *et al.*, 2018a; Toranj *et al.*, 2020). Thus, we speculate that there is a certain threshold of VP activity under nonstressed conditions for cell viability and plant growth. Moreover, pleiotropic adaption to the higher VP activity could take place in the stable transgenic plant lines, thus overcoming any otherwise potential negative effect on plant cell performance. However, above the tolerance threshold associated to a physiological context, the transiently increased VP activity becomes detrimental, even leading to cell death. Activation of plant resistance (R) protein in virus‐infected plants also can cause hypersensitive response (HR), leading to localized virus infection in plant (Hallwass *et al.*, 2014). Therefore, we propose that induction of TaVP accumulation restricts CWMV infection through an HR at the early stage of CWMV infection. Based on the expression pattern of *TaVP* after CWMV inoculation, we conclude that the accumulation of vsiRNA‐20 in CWMV‐infected wheat plants represses the transcription of *TaVP*, leading to an inhibition of cell death. Indeed, our results demonstrate that vsiRNA‐20 is capable of inhibiting HR in *N. benthamiana* leaves and this inhibition is vsiRNA‐20 dosage‐dependent (Fig. 8). Consistent with these results, we also discovered obvious cell death in the leaves of CWMVm‐inoculated wheat plants but not in the CWMV‐inoculated plants or TaVP‐silenced wheat plants at 20 dpi (Fig. 9). Cell death usually limits cell‐to‐cell movement of plant viruses in plant cell. Here, we speculate that vsiRNA‐20 promotes the CWMV replication in the plant cell. This speculation is supported by the fact that the accumulated level of CWMV(−) viral RNA was significantly decreased in the protoplast inoculated with CWMVm but not in the protoplast inoculated with CWMV (Fig. 7d).

**Figure 10:**
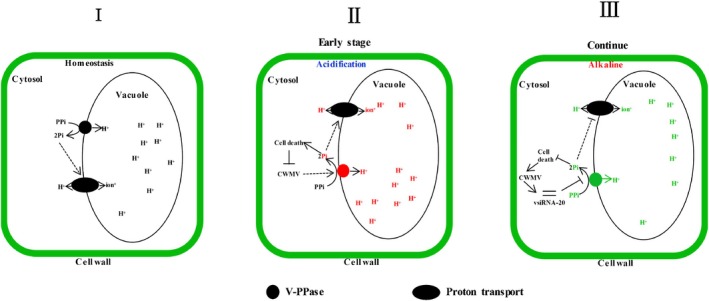
A working model for virus‐derived small interfering RNA‐20 (vsiRNA‐20) function in *Chinese wheat mosaic virus* (CWMV) infection in wheat. (I) An uninfected wheat cell. (II) A wheat cell at the early stage of CWMV infection. The CWMV infection upregulated the expression of *Triticum aestivum vacuolar (H^+^)‐PPase* (*TaVP*). The increased level of TaVP rapidly increased the number of operating pumps, changed the intracellular pH values, and altered the chemiosmotically derived cation/H^+^ exchange through membrane, leading to an acidified cytoplasm and cell death. This cell death restricts CWMV infection in localized areas. (III) As CWMV infection progresses, abundant vsiRNA‐20 will accumulate in the cytosol. The accumulated vsiRNA‐20 will target *TaVP* expression in cells to decrease the level of TaVP in tonoplast, leading a change of intracellular pH values and then alterations of chemiosmotically driven cation/H^+^ exchange through membrane. These changes will result in a weak alkalization in the cytosol to prevent cell death and promote CWMV accumulation in cells. Red, upregulated expression; green, downregulated expression.

In summary, we propose a working model for vsiRNA‐20 function in CWMV infection in wheat. We consider that VPs use energy generated through hydrolysis of PPi that is produced by ATP hydrolysis to promote active proton transport across membranes in order to support plant development and normal growth (Fig. 10I). During early stages of CWMV infection, the expression of VPs is significantly induced, leading to an HR and inhibition of virus infection (Fig. 10II). After CWMV has established its infection in wheat, large amounts of vsiRNA‐20 in cytoplasm specifically suppress the accumulation of VP mRNAs via a base pairing reaction. After *VP* expression is downregulated, less VP can be found in the tonoplast. As a result, the HR is suppressed and cytoplasm inside the CWMV‐infected cells becomes weakly alkaline, which promotes CWMV replication in the infected plants (Fig. 10III).

## Author contributions

JY and JC conceived the project and designed the experiments; JY carried out the experiments with assistance from NW, LH, JL, TZ, JinY, GW and XC; all authors analyzed and discussed the results; and JY and JC wrote the manuscript.

## Supporting information

Please note: Wiley Blackwell are not responsible for the content or functionality of any Supporting Information supplied by the authors. Any queries (other than missing material) should be directed to the *New Phytologist* Central Office.

**Fig. S1** Sequence alignment using deduced *T. aestivum* vacuolar (H^+^)‐PPase (TaVP) protein sequence and its known homologs.
**Fig. S2** Detection of *T. aestivum* vacuolar (H^+^)‐PPase fused to HA tag (TaVP‐HA) expression in *N. benthamiana* leaves.
**Fig. S3** Detection of virus‐derived small interfering RNA‐20 (vsiRNA‐20) expression level by qRT‐PCR.
**Fig. S4** Detection *Chinese wheat mosaic virus* (CWMV) infection in wheat plants inoculated with CWMV or the mutant of CWMV (CWMVm).
**Fig. S5** pH calibration.
**Table S1** Primers used for vector constructions and RT‐PCR analyses.
**Table S2** Detail information on *Chinese wheat mosaic virus* (CWMV)‐derived small interfering RNA (siRNAs) (reads of > 100).
