# Supplementary material for: Chinese wheat mosaic virus‐derived vsiRNA‐20 can regulate virus infection in wheat through inhibition of vacuolar‐ (H+)‐PPase induced cell death
Source: New Phytol. 2020 Jan 7;226(1):205–20. doi: 10.1111/nph.16358 (PMC7065157; doi:10.1111/nph.16358)
Supplement: Supplementary file 1 — Fig. S1 Sequence alignment using deduced T. aestivum vacuolar (H+)‐PPase (TaVP) protein sequence and its known homologs. Fig. S2 Detection of T. aestivum vacuolar (H+)‐PPase fused to HA tag (TaVP‐HA) expression in N. benthamiana leaves. Fig. S3 Detection of virus‐derived small interfering RNA‐20 (vsiRNA‐20) expression level by qRT‐PCR. Fig. S4 Detection Chinese wheat mosaic virus (CWMV) infection in wheat plants inoculated with CWMV or the mutant of CWMV (CWMVm). Fig. S5 pH calibration. Table S1 Primers used for vector constructions and RT‐PCR analyses. Table S2 Detail information on Chinese wheat mosaic virus (CWMV)‐derived small interfering RNA (siRNAs) (reads of > 100). [file NPH-226-205-s001.pdf]

New Phytologist

New Phytologist Supporting Information

Article title: *Chinese wheat mosaic virus*-derived vsiRNA-20 can regulate virus infection in wheat through inhibition of vacuolar- (H+)-PPase induced cell death

Authors: Jian Yang, Tianye Zhang, Juan Li, Ne Wu, Guanwei Wu, Jin Yang, Xuan Chen, Long He, Jianping Chen

Acceptance date: 22 November 2019

The following Supporting Information is available for this article:

Fig. S1 Sequence alignment using deduced *Triticum aestivum* Vacuolar (H^+)-PPase (TaVP) protein sequence and its known homologs. The identical amino acids are in black color. Names of the species are shown on the right side of the alignment: *Brachypodium distachyon* (Bd), *Hordeum vulgare* (Hv), *Oryza sativa* (Os), *Zea mays* (Zm) and *Triticum aestivum* (Ta).

Fig. S2 Detection of *Triticum aestivum* Vacuolar (H^+)-PPase fused to HA tag (TaVP-HA) expression in *N. benthamiana* leaves.

Fig. S3 Detection of virus-derived small interfering RNA-20 (vsiRNA-20) expression level by Quantitative reverse transcription (RT-qPCR). The ct value of vsiRNA-20 was detected in wheat plants infected with CWMV and wild type (WT) plants. Each relative transcripts level is presented as mean ± SD from three biological samples and each biological sample had three technical replicates. Statistical analyses were done using the Student's *t*-test. *, $P< 0.05$; n.s., no significant difference.

Fig. S4 Detection *Chinese wheat mosaic virus* (CWMV) infection in wheat plants inoculated with CWMV or the mutant of CWMV (CWMVm).

Detection CWMV infection in wheat plants inoculated with CWMV or CWMVm RNA transcript through reverse transcription PCR (RT-PCR) using CWMV *coat protein* (*CP*) gene specific primers. Total RNA from a buffer-inoculated wheat plant was used as a negative control (-). Diluted plasmid pCB-T7-R2 was used as a positive control (+).

Fig. S5 pH calibration.

The BCECF calibration curve was generated ex vivo by dissolving the free acid form of BCECF (2.5 µM) in a medium containing 1 Mm CaCl2 and 1 Mm MES, adjusted to the desired pH with Tris. Data points (n=3) were fitted with a linear fit (solid line) to convert ratio values into pH values.

Table S1 **Primers used in vector construction and molecular analyses**

Table S2 **Detail information on *Chinese wheat mosaic virus* (CWMV) derived small interfering RNAs (siRNAs) (reads of >100)**

Fig. S1

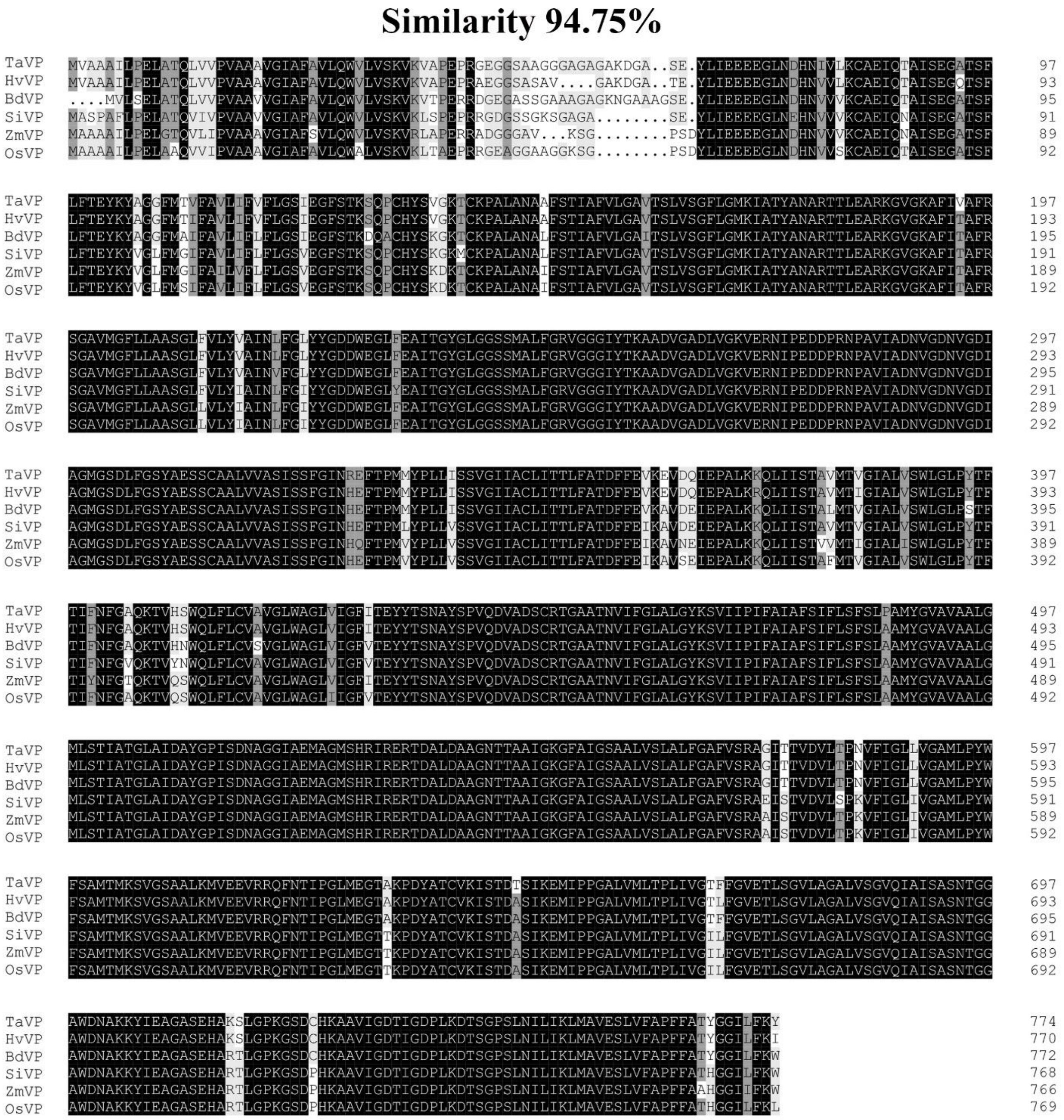

Fig. S2

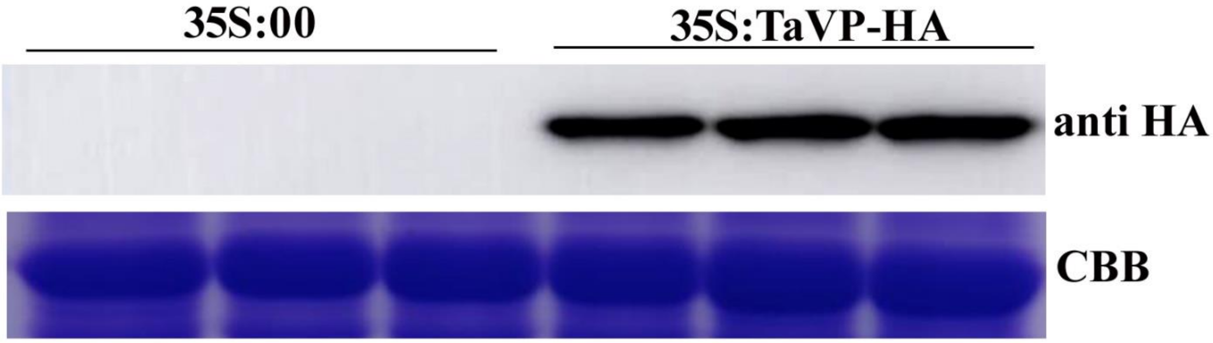

35S:00
35S:TaVP-HA
anti HA
CBB

Fig. S3

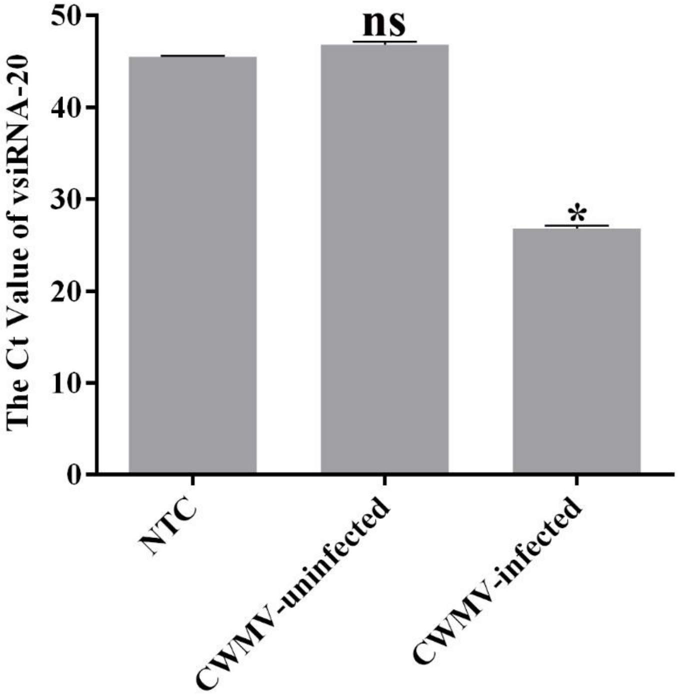

The Ct Value of vsiRNA-20
50
40
30
20
10
0
ns
*
NTC
CWMV-uninfected
CWMV-infected

Fig. S4

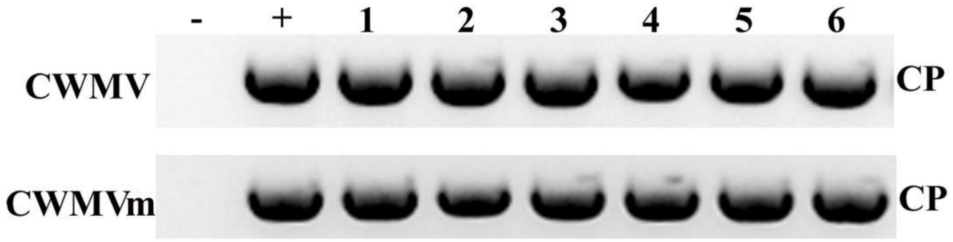

-
+
1
2
3
4
5
6
CWMV
CP
CWMVm
CP

Fig. S5

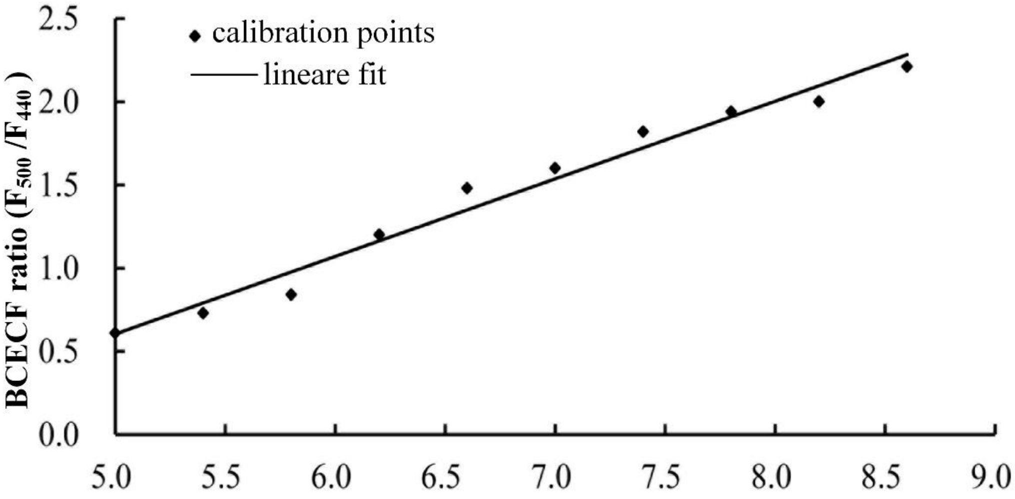

calibration points
lineare fit
BCECF ratio (F_{500} /F_{440})
2.5
2.0
1.5
1.0
0.5
0.0
5.0
5.5
6.0
6.5
7.0
7.5
8.0
8.5
9.0

Table S1

Primers	Primer sequences (5'-3')[a]	Position and description [b]	Usage
P1F	CGGACTAGTAGCATGAAGATCGACTATCTA	*SpeI*, 3'-UTR of *Ta*VP nt 2388-2410	p35S:GFP-UTR construction
P1R	ACGCGTCGACTGGCCGTTCATTTCGCTACCC	*SalI*, 3'-UTR of *Ta*VP nt 2586-2565	
P2F	CGCGGATCCTTTTGGCTGTAGCAGCAGCAG	*BamHI*	p35S:vsiRNA-20 construction
P2R	TTGGACGACGATGATGAATCTCTGCTG		
P3F	AGATTCATCATCGTCGTCCAACAGGAG		
P3R	AGATTCATCATCGTCGTCCAAAGAGAG		
P4F	TTGGACGACGATGATGAATCTCCTGCT		
P4R	CGAGCTCACAGAACAGCCTAGCAGCAGG	*SacI*	
P5F	CGCCATATGACAGAACAGCCTAGCAGCAGG	*NdeI*, partial sequence of *Ta*VP nt 60-81	pCaMV35S:*Ta*VP-GFP construction
P5R	CGGGATCCAGATACTTGAAGAGGATGCCT	*BamHI*, partial sequence of TaVP nt 2383-2362	
P6F	CCTTAATTAATTTGGGCTTTATTATGGTGAT	*PacI*, partial sequence of *Ta*VP nt 720-741	BSMV:*Ta*VP construction
P6R	ATTAAGAATGCGGCCGCTGAGGCAACAACAAGAG CAGC	*NotI*, partial sequence of *Ta*VP nt 974-953	
P7F	CGGACTAGTATGGGGCTCGATTTGGGAGCC	*SpeI*, ORF of *Ta*VP nt 158-179	p35S:*Ta*VP-HA construction
P7R	ACGCGTCGACACGGAGGCAGAAGACCGTAGT	*SalI*, ORF of *Ta*VP nt 1635-1614	
P8F	CGGGATCCGTATTTCTTTCTCTCTACGTC	*BamHI*, partial sequence of CWMV RNA1 nt 1-21	pCB-T7-R1M construction
P8R	CGACTCGTCGTCATCATCAAGGTCCTTCTCAAAATC AGCCCA	Partial sequence of CWMV RNA1 nt 2346-2325	
P9F	GAGTCTGGTTGGCGTTTCCATGGTGAC	Partial sequence of CWMV RNA1 nt 2367-2388	
P9R	CGAGCTCTGGGCCGGATAACCCTCCGGT	*SacI*, partial sequence of CWMV RNA1 nt 7145-7124	
P10F	TTTGGGCTTTATTATGGTGAT	Partial sequence of *Ta*VP nt 720-741	qRT-PCR of *Ta*VP
P10R	TGAGGCAACAACAAGAGCAGC	Partial sequence of *Ta*VP nt 974-953	
P11F	ACGAGTGTGTTGTCTCACCTT	Partial sequence of CWMV CP nt 121-142	Detection the CWMV CP
P11R	AGCATTAGATATAGCCAACGA	Partial sequence of CWMV CP nt 421-400	
P12F	GACTAAGGGATCATCTCAAGT	Partial sequence of BSMV CP nt 361-382	Detection the BSMV CP
P12R	TCCTTTAGATGTTTGTCATAC	Partial sequence of BSMV CP nt 661-640	
P13F	CGCGGATCCTTTTGGCTGTAGCAGCAGCAG	*BamHI*	p35S:vsiRNA-12 construction
P13R	TAGGCGTCGTTCAGTATCCAAGCTG		
P14F	TTGGATACTGAACGACGCCTACAGGAG		
P14R	TTGGATACTGAACGACGCCTAAGAGAG		
P15F	TAGGCGTCGTTCAGTATCCAACTGCT		
P15R	CGAGCTCACAGAACAGCCTAGCAGCAGG	*SacI*	
P16F	GGGGACAAGTTTGTACAAAAAAGCAGGCTGCATG GCTCATTCTCATGAAATG		p35S:OsVPE2-RFP construction
P16R	GGGGACCACTTTGTACAAGAAAGCTGGGTCCACCT CATCTGAGTAGGTACC		
attB1	GGGGACAAGTTTG TACAAA AAAGCAGGCTGC		
attB2	GGGGACCACTTTGTACAAGAA AGCTGGGTC		

*Ta*actinF	CTAACTGACTGTTTGATGAAG	Partial sequence of *Ta*actin nt 301-322	qRT-PCR of *Ta*actin
*Ta*actinR	CTTCATGATAGAGTTGTAGGT	Partial sequence of *Ta*actin nt 600-579	
*Nb*actinF	GAGACATTCAACGTTCCGGCT	Partial sequence of *Nb*actin nt 121-142	qRT-PCR of *Nb*actin
*Nb*actinR	CTCGTAGTCA AGAGCCACATA	Partial sequence of *Nb*actin nt 420-399	
GSP	CGACCATCTAGTCACCTACAG	Partial sequence of TC371364	5' RACE of TC371364
AP	CTAATACGACTCCACTATAGGGC	5'RACE adaptor	
Primer 1	ACCCTCCAGCATACTTGTACT	Partial sequence of *Ta*VP nt 382-361	RT-PCR of *Ta*VP
Primer 2	ACGGCGCCAGCGAGTACCTCA	Partial sequence of *Ta*VP nt 241-262	
Oliga DT	TTTTTTTTTTTTTTTTTTTTTTTTT		
GFPF	AATACAACTATAACTCACACA	Partial sequence of GFP nt 425-446	qRT-PCR of *GFP*
GFPR	GACAGGTAATGGTTGTCTGGT	Partial sequence of GFP nt 605-584	
P17F	AGAAAAACTCGTGCTATTCAG	Partial sequence of CWMV RNA1 genomic nt 6,541-6,562	qRT-PCR of viral RNA
P17R	GGACTAGAAGATAGTCGTACA	Partial sequence of CWMV RNA1 genomic nt 7020-6999	
P18F	CGAGCGTGT AGATTCATC		qRT-PCR of vsiRNA-20
P18R	CAGTGCAGGGTCCGAGGTATT		
Stem-loop primer	GTCGTATCCAGTGCAGGGTCCGAGGTATTCGCACTGGATACGAC TTGGACGAC		

Note: a, Underlined letters indicate restriction enzyme sites, b, Numbers correspond to target nucleotide positions; a reverse order of numbers indicates that the primer is complementary to the targeted sequences.

Table S2

vsiRNA ID	Reads	Sequences of vsiRNA (5'-3')	Genome segments	Size (nt)	virus blast (star)	virus blast (end)	Genomic/anigenomic strand	Mismatch number(≤1)
vsiRNA-1	235	ttcctgttaaatgtcgtctgc	CWMV_RNA2	21	2756	2776	+	0
vsiRNA-2	230	atacccaggtcgtcagcaagt	CWMV_RNA2	21	918	938	-	0
vsiRNA-3	192	aaaagagagatgttagtcggt	CWMV_RNA1	21	1762	1782	+	0
vsiRNA-4	172	gtttggttcctgttaaatg	CWMV_RNA2	19	2750	2768	+	0
vsiRNA-5	162	actaaaggctgactcgtcgct	CWMV_RNA1	21	2655	2675	-	0
vsiRNA-6	158	acttcaagtcgttggcgcata	CWMV_RNA1	21	1504	1524	+	0
vsiRNA-7	155	tgtattttttcgtcgacattg	CWMV_RNA2	21	3063	3083	-	0
vsiRNA-8	146	ggtttggttcctgttaaatg	CWMV_RNA2	20	2749	2768	+	0
vsiRNA-9	141	aagatacccaggtcgtcagca	CWMV_RNA2	21	921	941	-	0
vsiRNA-10	135	aaaaatggttggacaagcaaa	CWMV_RNA2	21	1619	1639	+	0
vsiRNA-11	134	tttggttcctgttaaatgtcg	CWMV_RNA2	21	2751	2772	+	0
vsiRNA-12	131	ttggatactgaacgacgccta	CWMV_RNA2	21	2460	2480	-	0
vsiRNA-13	117	tggtgagggtaggacgccggtt	CWMV_RNA2	22	288	309	+	0
vsiRNA-14	117	aaagaaataaaatattctcggg	CWMV_RNA2	22	66	87	+	0
vsiRNA-15	115	agatacccaggtcgtcagcaa	CWMV_RNA2	21	920	940	-	0
vsiRNA-16	110	agaacaagggcagactgacca	CWMV_RNA2	21	702	722	+	0
vsiRNA-17	105	tacccaggtcgtcagcaagtg	CWMV_RNA2	21	917	937	-	0
vsiRNA-18	101	cgagactgatctgttgttgag	CWMV_RNA1	21	401	421	+	0
vsiRNA-19	100	gtattttttcgtcgacattga	CWMV_RNA2	21	3062	3082	-	0
vsiRNA-20	100	agattcatcatcgtcgtccaa	CWMV_RNA1	21	2346	2366	-	0
vsiRNA-21	100	taagatacccaggtcgtcagc	CWMV_RNA2	21	922	942	-	0
vsiRNA-22	100	attttttcgtcgacattgaa	CWMV_RNA2	20	3060	3079	-	0
